# New Insights on the Sialidase Protein Family Revealed by a Phylogenetic Analysis in Metazoa

**DOI:** 10.1371/journal.pone.0044193

**Published:** 2012-08-30

**Authors:** Edoardo Giacopuzzi, Roberto Bresciani, Roland Schauer, Eugenio Monti, Giuseppe Borsani

**Affiliations:** 1 Department of Biomedical Sciences and Biotechnology, Unit of Biology and Genetics, University of Brescia, Brescia, Italy; 2 Department of Biomedical Sciences and Biotechnology, Unit of Biochemistry and Clinical Chemistry, University of Brescia, Brescia, Italy; 3 Institute of Biochemistry, Christian-Albrechts University, Kiel, Germany; University of Georgia, United States of America

## Abstract

Sialidases are glycohydrolytic enzymes present from virus to mammals that remove sialic acid from oligosaccharide chains. Four different sialidase forms are known in vertebrates: the lysosomal NEU1, the cytosolic NEU2 and the membrane-associated NEU3 and NEU4. These enzymes modulate the cell sialic acid content and are involved in several cellular processes and pathological conditions. Molecular defects in NEU1 are responsible for sialidosis, an inherited disease characterized by lysosomal storage disorder and neurodegeneration. The studies on the biology of sialic acids and sialyltransferases, the anabolic counterparts of sialidases, have revealed a complex picture with more than 50 sialic acid variants selectively present in the different branches of the tree of life. The gain/loss of specific sialoconjugates have been proposed as key events in the evolution of deuterostomes and *Homo sapiens,* as well as in the host-pathogen interactions. To date, less attention has been paid to the evolution of sialidases. Thus we have conducted a survey on the state of the sialidase family in metazoan. Using an *in silico* approach, we identified and characterized sialidase orthologs from 21 different organisms distributed among the evolutionary tree: Metazoa relative (*Monosiga brevicollis),* early Deuterostomia, precursor of Chordata and Vertebrata (teleost fishes, amphibians, reptiles, avians and early and recent mammals). We were able to reconstruct the evolution of the sialidase protein family from the ancestral sialidase NEU1 and identify a new form of the enzyme, NEU5, representing an intermediate step in the evolution leading to the modern NEU3, NEU4 and NEU2. Our study provides new insights on the mechanisms that shaped the substrate specificity and other peculiar properties of the modern mammalian sialidases. Moreover, we further confirm findings on the catalytic residues and identified enzyme loop portions that behave as rapidly diverging regions and may be involved in the evolution of specific properties of sialidases.

## Introduction

Sialidases or neuraminidases comprise a family of glycohydrolytic enzymes that remove sialic acid residues from various sialo-derivatives, such as glycoproteins, glycolipids (gangliosides) and oligosaccharides [1]. These exoglycosidases are widely distributed in nature, including viruses, protozoa, bacteria, fungi and vertebrates [2]. Most of the sialidases with a defined 3D architecture, such as various proteins from viruses and bacteria and the human cytosolic sialidase NEU2, show a typical 6 blade β-propeller structure [3]. They are mainly classified in CAZy (Carbohydrate Active Enzymes database) families GH33 (bacterial and eukaryotic enzymes), GH34 (influenza viruses enzymes), GH83 (other viral sialidases) and GH58 (bacteriophage endosialidases) [4]. The various sialidases show the presence of a conserved T/FYRI/VP motif in the N-terminal region, together with a variable number of Asp-box motifs [1]. Strictly essential catalytic residues are highly conserved and comprise: 3 Arg, that bind the carboxylate group common to all sialic acids, a Tyr/Glu nucleophile pair and an Asp that acts as the acid/base catalyst [3]. In vertebrates the protein family comprises four different forms: the lysosomal sialidase NEU1, the soluble or cytosolic sialidase NEU2 and the membrane-associated sialidases NEU3 and NEU4 [1]. NEU1 has been the first member of the family to be identified and the most studied, since its dysfunction is responsible for two genetic diseases, sialidosis (OMIM #256550) and galactosialidosis (OMIM #256540). Reduced enzyme activity in the lysosomes causes accumulation of gangliosides and other sialylated molecules that results in severe cytotoxicity and cell death. Symptoms include neurodegeneration, reduced mobility and visual impairment. The other members of the sialidase family are also key elements in important cellular processes, although there are no evidences of a direct involvement in pathogenic conditions. Briefly, NEU2 is involved in myoblast differentiation, NEU3 in cell differentiation, signal transduction modulation, cell-to-cell interactions and tumorigenesis, while NEU4 participates in apoptotic processes and neuronal differentiation [1]. In general, the importance of sialidases resides in their ability to modify the complex patterns of sialylated molecules in the intracellular environment as well as on the cell surface, thus providing a fine regulation of sialic acid content [2].

Sialic acid residues are detected mainly in the deuterostome lineage (vertebrates, ascidians and echinoderms). They are usually attached at the terminus of glycan chains on the cell surface and can be subjected to numerous modifications generating more than 50 structurally distinct molecules [5], [6]. Such a wide diversity is an outcome of evolutionary selection by host-pathogen interactions acting along with pressure to conserve critical endogenous functions. Indeed, glycoconjugates on the cellular surface are known to affect different biological events including tumorigenic transformation, cell differentiation and motility [7], [8] and regulation of the immune response [9]. Further confirming the pivotal role played by sialic acids, expression of some of the genes involved in the biochemistry of sialylated glycans are altered in cancer, injury and inflammation, resulting in changes in the glycan patterns. Sialic acids are also critical in the development of vertebrates and play a main role in human evolution [10], [11]. Today 60 genes are known to be directly involved in sialic acid biology and among them at least 10 show unique genetic changes in humans when compared with our closest evolutionary relatives [12]. These modifications resulted in tissue-specific changes of cell surface glycosylation, that in turn caused adaptations in various endogenous proteins that interact with these glycans, as well as in pathogen recognizing them.

Given the critical roles of the various classes of sialic acids, a number of studies have been carried out to elucidate their diffusion among different multicellular organism as well as in several pathogenic microorganisms [6], [10]. Phylogeny and function of the main enzymes connected to the synthesis of sialoconjugates have also been studied and recently an extensive survey has been conducted for sialyltransferases (ST), reconstructing their state in Deuterostomia and Protostomia [13], [14].

On the other hand, relatively little attention has been paid to the catabolism of sialoconjugates, namely the degradative pathway starting with the removal of sialic acids by the sialidase enzymes. Indeed, besides viruses and bacteria, sialidases have been deeply studied only in mammals, with 11 mammalian sialidases cloned and described so far, each one with different subcellular localization and substrate specificities [15]. Only recently this class of enzymes has been studied also in lower vertebrates, such as the teleost zebrafish [16], and in birds [17]. In this perspective we reconstructed the phylogeny of sialidase enzymes in Metazoa, from their closest living relative *M. brevicollis,* through early Deuterostomia (Echinoidea) and Chordata, up to Vertebrata and Mammalia.

## Materials and Methods

### Sialidase Sequences Identification

We selected for this study 21 organisms representative of the main taxa in Eukaryota and chosen based on availability of genomic information and their position at key points in Eukaryota evolutionary tree (a representation of their placement in the evolution is given in Figure S1 and the scientific names, together with common ones, are given in Table 1). Orthologous sialidase proteins were identified from protein, genomic and EST sequences available in central repositories such as NCBI, ENSEMBL or DDBJ or in specialized databases, such as those available at the Joint Genome Institute (JGI) for the amphioxus *B. floridae* and the Choanoflagellate *M. brevicollis* and at the Baylor College of Medicine for the acorn worm *S. kowalevskii.* Searches were performed using TBLASTN and PSI-BLAST with default parameters (an e-value cut off at 0.01 was used in all BLAST searches). Databanks survey was completed in November 2011. Human sialidase protein sequences were used as queries in the first round of searches and the retrieved sequences were subsequently used to improve searches in lower organisms. Assembly of contigs from the different ESTs of each gene and additional manipulation of retrieved sequences were made with Invitrogen Vector NTI v.10. The complete open reading frames identified where analyzed for the presence of typical sialidase motifs (e.g. T/FYRI/VP motif and Asp boxes) and then submitted to GenBank. Only sequences allowing generation of a protein containing all the residues essentials for the catalytic domain were considered in subsequent analysis. When the assembled coding DNA sequences (CDS) resulted incomplete, we tried to extended them using a gene prediction software (GENSCAN Web Server at MIT).

**Table 1:** Scientific names, common names and three letter code abbreviations used for the 21 organisms considered in the study.

Scientific name	Common name	Abbreviation
*Homo sapiens*	Human being	Hsa
*Equus caballus*	Horse	Eca
*Bos taurus*	Cattle	Bta
*Mus musculus*	House mouse	Mmu
*Monodelphis domestica*	Gray short-tailedopossum	Mdo
*Ornithorhynchus anatinus*	Platypus	Oan
*Gallus gallus*	Red junglefowl chicken	Gga
*Taeniopygia guttata*	Zebra finch	Tgu
*Anolis carolinensis*	Green anole lizard	Aca
*Xenopus tropicalis*	Western clawed frog	Xtr
*Danio rerio*	Zebrafish	Dre
*Petromyzon marinus*	Sea lamprey	Pma
*Branchiostoma floridae*	Lancelet	Bfl
*Saccoglossus kowalevskii*	Acorn worm	Sko
*Strongylocentrotus* *purpuratus*	Purple sea urchin	Spu
*Paracentrotus lividus*	Purple sea urchin	Pli
*Drosophila melanogaster*	Fruit fly	Dme
*Oscarella lobularis*	Blue sponge	Olo
*Monosiga brevicollis*	–	Mbr
*Karenia brevis*	–	Kbr
*Spironucleus vortens*	–	Svo

### Subfamily Assignment and Residues Analysis

Putative orthologs were initially identified and assigned to subfamilies using a reciprocal best BLAST hits approach [18]. The subgroup assignment of each hypothetical enzyme was further confirmed by the analysis of the pairwise similarity matrix, its position in the calculated phylogenetic tree (see below) and the visual inspection of the sequence. The estimates of evolutionary divergence between each subgroups was calculated with MEGA5 [19] taking into account the number of amino acid substitutions per site from averaging over all sequence pairs between subgroups. This analysis was conducted using a Maximum Likelihood method and the WAG matrix-based model for amino acid substitutions [20], with the rate variation among sites modeled with a gamma distribution (shape parameter = 5). Only positions with more than 70% site coverage were considered informative. Subfamily logo [21], generated by TexShade package [22], was used to identify residues showing peculiar conservation in the single subgroups. NEU1 and NEU5 subgroups were compared each one versus NEU2, 3 and 4 sequences taken together. NEU2, NEU3 and NEU4 subgroups were compared each one against the other two taken together.

### Multiple Alignments, Phylogeny and Exon Structure

Protein sequences were aligned with MUSCLE v3.7 with default parameters (the complete alignment in FASTA format is provided as Multiple FASTA alignment S1) and the multiple alignments were visualized using TexShade, a Latex package [22]. Phylogenetic analysis was automated using the MEGA5 software [19]. First, 6 different amino acid substitution models with or without empirical frequencies, invariant sites and the gamma model for rate variation (for a total of 48 evolutionary models), were evaluated on our dataset according to Bayesian Infromation Criterion (BIC). WAG model [20] with Gamma distributed evolutionary rate among sites, but without invariant sites and empirical frequencies (WAG+G) resulted as the best model (lowest value of BIC). Phylogenetic trees were then calculated with MEGA5, using a Maximum Likelihood (ML) analysis based on the WAG+G model of amino-acid substitution and with 1000 bootstrap replicates. The estimated value of the shape parameter for the discrete Gamma Distribution with 5 categories is 2.6845. Only positions with at least 50% sequence coverage were considered as informative in phylogeny reconstruction, resulting in a final dataset with a total of 379 positions. Initial tree(s) for the heuristic search were obtained automatically by applying Neighbor-Join and BioNJ algorithms and then selecting the topology with superior log likelihood value. To infer NEU1 evolution, computation of gene coalescence within species tree was carried out using Mesquite modules [23]. The phylogenetic tree of the 21 species considered, obtained from NCBI taxonomy data, was used as primary tree and the subtree containing NEU1 sequences, extracted from the global tree of sialidases calculated with MEGA5 as described above, was used as the inner tree for the gene coalescence analysis.

For intron-exon structure determination we used, when available, previously annotated gene models from public databases. For newly assembled sialidase CDS we used Wise2 from EBI (http://www.ebi.ac.uk/Tools/Wise2/), together with UCSC Genome Browser Blat or BLAST services provided at the specific genome project portals, when a complete genomic sequence was publicly available. The corresponding protein sequence was used to determine the exon organization of the coding portion. The reciprocal positions of the various exon junctions within the sialidase polypeptides was then evaluated in the global multiple alignment containing all the identified sialidase protein sequences (FASTA alignment provided as Multiple FASTA alignment S1).

### Analysis of Phosphorylation Sites

Phosphorylation sites were predicted for each one of the human sialidase NEU1–4 and for the *B. floridae* NEU5 using NetPhos 2.0 [24] and the conservation of the positions identified were analyzed in the multiple alignment containing all the 83 sialidase sequences (Multiple FASTA alignment S1). In every single sequence, a position was considered as phosphorylation site if one of the three Ser, Thr or Tyr residue occurs, independently from which one was predicted by NetPhos in the query sequence. The significance of the conservation of phosphorylation sites in the complete alignment was assessed using a binomial statistical test where k was equal to the number of S, T and Y residues in the alignment column; N was equal to the total number of residues in that column (83); p was equal to the frequency of S, T and Y residues overall the entire alignment (0.023 over 87696 sites). Specific conservation in the 5 sialidase subgroups was also evaluated using the Fisher’s exact test. For every subfamily, we constructed a 2-by-2 contingency table with rows representing the subfamily of interest and the other 4 subfamilies taken together, and columns representing the phosphorylatable (S, T and Y) residues and the non phosphorylatable residues occurring at the considered position. Count of the residues in each of the 4 category was then used in the test. Conservation of phosphorylation positions in specific evolutionary subgroups was evaluated grouping the 83 sequences in four categories based on taxonomic classification: three nested groups, mammals, vertebrates and deuterostomes; plus the outer group of early Metazoa. Significance of specific conservation in these taxonomic groups was assessed using the Fisher’s exact test on a 2-by-2 contingency table constructed following the same approach applied for the specific conservation in sialidase subgroups. Results from binomial and Fisher statistical tests were corrected using Bonferroni correction to account for multiple hypothesis testing. Within the results obtained from statistical filtering, we considered relevant only those showing ≥60% sequence conservation in at least one of the defined groups.

### Synteny Analysis and Co-evolution with Sialyltransferases

Synteny between human sialidases and related genes in other organisms was assessed using Genomicus, an automated web service based on Ensembl dataset (version 61) that allows also the identification of paralogous blocks.

Sialyltransferases (STs) distribution among considered organisms was reconstructed in Deuterostomia and sponges based on phylogenetic studies on this class of enzymes [13], [14] and complemented with data from the CAZy database (GlycosylTransferase family 29). Putative STs sequences from lower organisms were retrieved using the same reciprocal BLAST approach applied for sialidases, with human STs protein sequences as queries (ST3Gal1 NP_003024; ST6Gal1 NP_003023; ST6GalNAc1 NP_060884; ST8Sia1 NP_003025). Using this approach we identified putative ST3Gal sequences in protists (GE181105 from the Coccolithophores *Emiliana huxleyi;* GW809693 from the red tide dinoflagellate, *Alexandrium minutum*), but failed to retrieve significant matches in *M. brevicollis.* For the aim of this study we considered the appearance of a new class of enzymes within the family as a new character, resulting in 5 characters for sialidases (NEU1–4 and NEU5) and 4 for STs (ST3Gal, ST6Gal, ST6GalNac, ST8Sia). An additional character was introduced for STs accounting for the presence of the complete set of enzyme subclasses in Mammalia.

### Molecular Modelling

The complete structure of human sialidase NEU2 in complex with the competitive inhibitor DANA [25] was retrieved from RCSB Protein Data Bank (PDB) [26] under accession 1vcu and used as template for all protein models. Structural models were constructed using I-Tasser [27] with NEU2 as template and standard parameters for human NEU1, NEU3 and NEU4. Members of NEU1 subgroup from *M. brevicollis*, *O. lobularis*, *S. purpuratus*, *B. floridae* and *P. marinus* and members of the newly identified NEU5 subgroup from *S. purpuratus*, *B. floridae* and *P. marinus* were also modeled using the same approach. The PDB structures obtained were visualized, aligned and manipulated using PyMol (The PyMOL Molecular Graphics System, Version 1.3, Schrodinger, LLC).

## Results

When non otherwise specified residues are indicated with coordinates referred to the human NEU2 protein sequence (NP_005374.2).

### Bioinformatic Analysis Allowed to Identify 87 Sialidase Sequences in 21 Organisms and to Define the New NEU5 Subgroup

By applying the bioinformatic strategy described in Material and Methods we were able to retrieve a total of 87 putative sialidase sequences. Detailed informations for each sequence are provided in Table S1. Based on the analysis performed, these sequences were initially divided in 4 subgroups: 40 sequences were marked as NEU1-like, 8 as NEU2-like, 20 as NEU3-like, 11 as NEU4-like. Multiple alignments, inspection of the protein sequence features and analysis of the intron-exon structure, allowed us to define a fifth subgroup, named NEU5, which in BLASTp queries obtained similar scores against NEU3 and NEU4 mammalian proteins and contains 8 sequences: 2 from the sea urchins investigated (*P. lividus, S. purpuratus*) and the lamprey (*P. marinus*), and 1 from the acorn worm (*S. kowalevskii)* and the amphioxus (*B. floridae)*. Interestingly, there no one of the considered organism showed the presence of all 5 sialidase subgroups. We then filtered out 4 incomplete sequences that produced poor alignments, namely NEU1 from chicken (*G. gallus),* one of the identified NEU1 sequences in the protozoan *S. vortens,* NEU1 and NEU3 from platypus (*O. anatinus*). Subsequent analysis were conducted on a total of 83 sequences. We were unable to identify sialidase-like sequences in the fruit fly (*D. melanogaster)* as well as in other organisms belonging to Protostomia whose sequences are available in public databases. The mean estimates of evolutionary divergence over sequence pairs between each subgroup calculated as described in Material and Methods are reported in Table 2.

**Table 2:** Estimates of evolutionary divergence over sequence pairs between subgroups.

	NEU2s	NEU3s	NEU4s	NEU5s
**NEU1s**	2.62 (0.17)	2.59 (0.16)	2.51 (0.16)	2.53 (0.16)
**NEU2s**		1.19 (0.09)	1.09 (0.09)	1.76 (0.14)
**NEU3s**			1.09 (0.08)	1.71 (0.12)
**NEU4s**				1.72 (0.13)

Standard error estimates are given in brackets.

### Specific Aminoacid Changes are found in each Sialidase Subgroups, While Essential Sialidase Features Remain Highly Conserved Throughout Evolution

**Figure 1:**
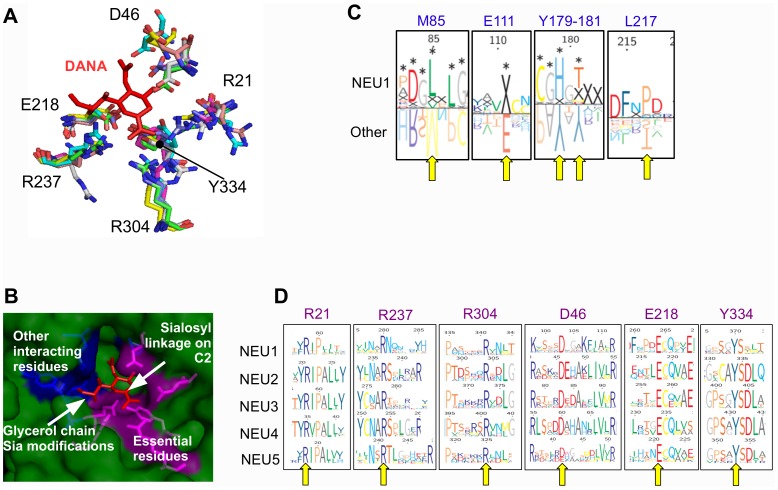
Analysis of sialidase catalytic crevice. (A) The superimposition of the structural models obtained for sialidase enzymes showed that the localization of the 6 key residues (those essentials for catalysis) within the protein structure is well conserved. The enzyme competitive inhibitor DANA is depicted in red. (B) Surface representation of the catalytic crevice in human NEU2 structure. Key residues, in magenta, are located near the C1 carboxyl group and the C2 (hemiketal carbon of Sias involved in the formation of the sialosyl linkage), while other residues interacting with the substrate, in blue, interact with the C-4 hydroxyl group, C-5 acetamido group and glycerol side-chain of DANA, where most of the natural Sia modifications occur. (C) Analysis of the 5 amino acid residues interacting with the competitive inhibitor DANA but not essential in the catalytic process, as identified from human NEU2 crystal structure. Subfamily LOGO analysis comparing NEU1 subgroup with other sialidase subgroups revealed specific differences on these 5 residues. X indicate missing residues and significant differences are marked with *. L217 shows no subgroup specific conservation in this comparison. (D) LOGO representations of the multiple alignments of the five sialidase subgroups show high sequence conservation for all the 6 key residues involved in the catalytic process. Yellow arrows in (C) and (D) indicate the considered residues. Residues are numbered according to human NEU2 protein sequence. The same color code is used in panels (B), (C) and (D) to indicate the residues that coordinate DANA.

**Figure 2:**
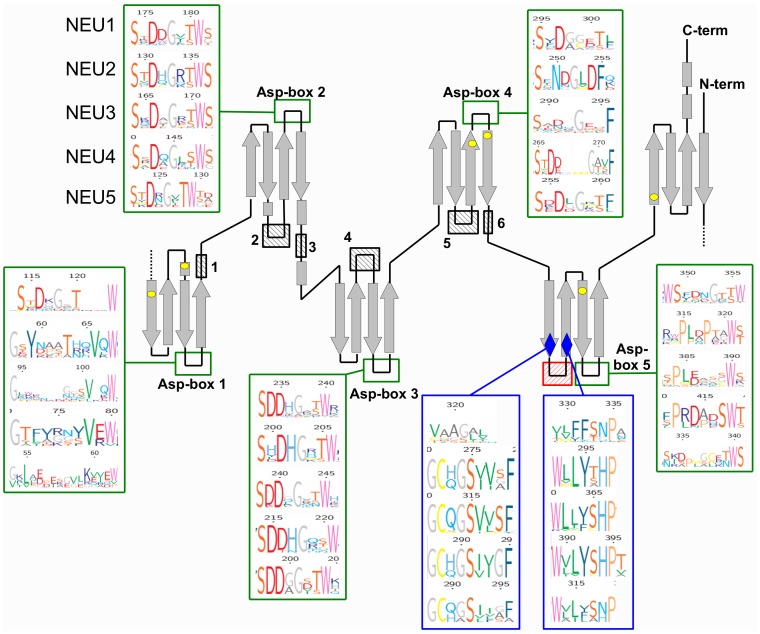
Schematic representation of the sialidase structure. β-sheets are represented as grey arrows, loops are depicted as black lines and the positions of the 6 key residues involved in the catalysis are indicated with yellow dots. The five Asp-boxes sites (Asp 1–5) are indicated with a green square. LOGOs representing sequence conservation in each one of the 5 sialidase subgroups are reported for each Asp-box. The black crossed boxes (1–6) indicate the loops in which large functional domains have been identified in leech, bacterial or viral sialidases. Number legend: 1: *M. decora*; 2: *C. perfrigens*, *S. pneumoniae*; 3: *V. cholerae*; 4: *C. perfringens*, *Newcastle* virus, *M. decora*; 5: *S. tiphimurium*; 6: i*nfluenza B* and i*nfluenza N9* viruses. The red crossed box indicates the highly variable loop identified in vertebrates, surrounded by two highly conserved sequence blocks, represented by blue diamonds. For these blocks LOGOs representation of the multiple alignments in the 5 sialidase subgroups are also reported.

**Figure 3:**
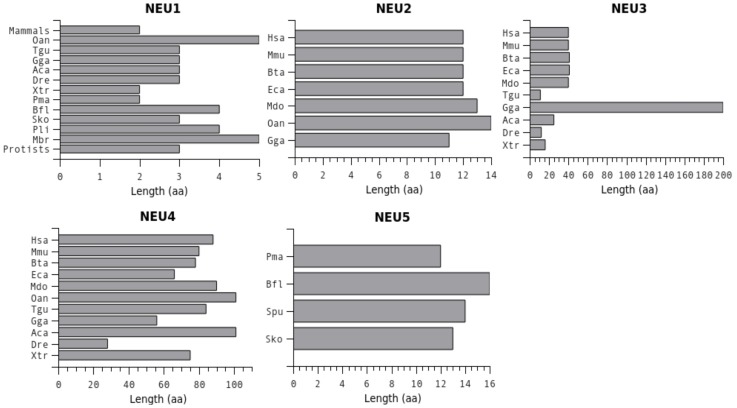
Length of the highly variable loop connecting the first and second strand of the fifth blade of the β-propeller. Length of the highly variable loop connecting the first and second strand of the fifth blade of the β-propeller, identified in sialidases from vertebrates (red crossed box in Figure 2). The loop is considerably shorter in NEU1 and shows the greater length variability in NEU3 and NEU4 enzymes.

**Figure 4:**
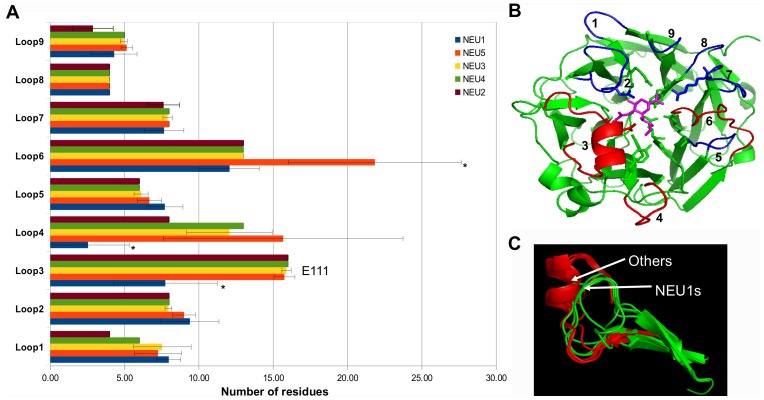
Analysis of the 9 loops emerging on the same side of the active site. (A) Horizontal bar chart representation of the mean length of the single loops in the 5 sialidase subgroups. Significant differences in length occur in Loop 3, 4 and 6 and are indicated with *. (B) Top view of the human NEU2 structure showing the 9 loops analyzed. Loops with significant length variations are near the glycerol portion of DANA and are represented in red, other loops are in blue. Catalytic key residues are represented as sticks and DANA inhibitor is colored in magenta. (C) Structural representation of Loop 3, containing the E111 residue, in different sialidases. Comparison of structural models revealed that this loop is considerably shorter in NEU1s than in other sialidases. The short α-helix structure containing E111 residue is absent in NEU1s. The positions of each loop within the human NEU2 structure are: Loop 0 (aa 16–18), Loop 1 (aa 15–18), Loop 2 (aa 42–49), Loop 3 (aa 107–122), Loop 4 (aa 183–190), Loop 5 (aa 238–243), Loop 6 (aa 261–274), Loop 7 (aa 299–306), Loop 8 (aa 329–332), Loop 9 (aa 357–359).

**Figure 5:**
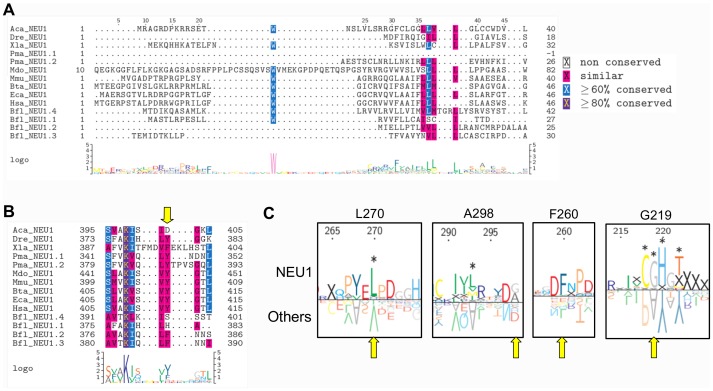
Analysis of NEU1 specific features. Multiple alignment of NEU1 protein sequences revealed that the signal peptide present at the N-terminal in the mammalian enzymes is poorly conserved in vertebrates (A), while the lysosomal localization signal at C-terminus is still present throughout Chordata (B). The conserved aromatic residue, usually placed at −4 position, is indicated by the yellow arrow in (B). (C) LOGO representation of the 4 residues essential for the interaction between NEU1 and PPCA. These residues are highly conserved in the NEU1 subgroup but they are lost in other sialidases. Residue numbering is according to human NEU1 sequence.

For each one of the five sialidase subgroups, protein sequences were aligned using MUSCLE and resulting multiple alignments were visualized using TexShade (see Subgroup alignments S1). Analysis of these data confirmed a complete conservation for all the 6 residues essential for the catalytic process, namely the Arg triad (R21, R237 and R304), the Tyr/Glu nucleophile pair (Y334 and E218) and an Asp acting as the acid/base catalyst (D46) (LOGO rapresentation in Figure 1D; structural representations in Figure 1A, B) [3], [25], [28]. By visual inspecting the alignment, also Asp-box motifs result overall conserved among different sialidases. Asp-boxes 2 and 3 showing the highest degree of conservation across all sialidase subgroups, while Asp-box 1 and Asp-box 5 appear fully conserved only in the NEU1 subgroup (Figure 2). Interestingly, in NEU5 these latter Asp-boxes are fairly conserved. All the other loops connecting the various β-sheets in the sialidase structures are highly divergent in sequence and comparison with known structures from viral and microbial sialidases revealed that they can host large insertions (Figure 2). Noteworthy, the loop located between the first and the second β-sheet of the fifth blade is highly variable in size (roughly from 3 to 200 residues) among vertebrates (Figure 3). Analysis of the loops located around the enzyme catalytic crevice, numbered 1–9 from N- to the C-terminus of the polypeptide, revealed that loops 3 and 4 are significantly shorter in NEU1 subgroups, while loop 6 is longer in NEU5 subgroup (Figure 4A, B). The protein portion containing the E111 residue (Loop 3) revealed also interesting structural features (Figure 4C), as detailed below. Residues specific for every subgroup identified from the subfamily logo analysis [21] are reported in Table 3. Analysis of the subfamily logo comparing NEU1 subgroup versus the other subgroups at the position of the 5 residues known to interact with the substrate from structural analysis of NEU2 [25], [28] revealed significant differences for M85, E111, Y179 and Y181, while residue L217 does not show a specific conservation out of the NEU2 subgroup (Figure 1C). Residues involved in the interaction with Cathepsin-A (CTSA or PPCA) as defined in [29] are also conserved specifically in NEU1 subgroup (Figure 5C). Surprisingly, other residues whose mutations result in the inactivation of the NEU1 enzyme [29] show no significant conservation in sialidase sequences (data not shown). Finally, multiple alignment of NEU1 sequences revealed that both the N-terminal signal peptide (aa 1–45 in *H. sapiens*) and the C-terminal sequence signal Y(-4) [30] are conserved only in higher organisms. The first seems conserved only in mammals (Figure 5A), while the aromatic residue seems retained through Chordata phylum (Figure 5B).

**Table 3:** Amino acid differences revealed by subfamily LOGO analysis comparing the 5 sialidase subgroups.

Sialidase subgroup	Specific residues
**NEU1**	W65, F77, A110, S114, D130, P134, D135, G136, L137, L139, G140, A141, Y155, S156, S158, H160, N161, Q165, S168, T169, M170, N186, S188, P192, T193, E194, M195, Y202, K212, C218, G219, H220, T222, D226, L231, L270, V276, I277, N281, L293, L302, L313, V318, A319, A320, A322, S326, V329, F331, F332, N334, T345, H347, R348, W349, S351, R357, T360, S372, L389, V391, K395, K408, I409
**NEU2**	G16, A19, I52, L115, L165, R172, S173, P190, S193, L234, N250, T325, A328, Q338, M340, Q371, F373
**NEU3**	E35, I59, F71, D85, T119, E127, S130, C139, I162, Y215, G241, I264, S276, A285, E286, Q313, V316, N381, F419, C421, C427
**NEU4**	M13, V36, H62, T121, L185, H192, L237, S239, Q262, D267, H289, G294, M404, R407, S409, I424, Y425, Y451
**NEU5**	E92, L223, E245, F294, C355

Numbering is referred to the corresponding human sialidase protein sequence. When a position shows multiple residues within the same subgroup, the most frequent is reported in the table. Hydrophilic amino acids, likely to be exposed on the surface of the protein structure, are underlined.

### All Sialidases Subgroups Show a High Conservation of the Exon Structure, with the Exception of NEU5 and Sequences from *M. brevicollis*

**Figure 6:**
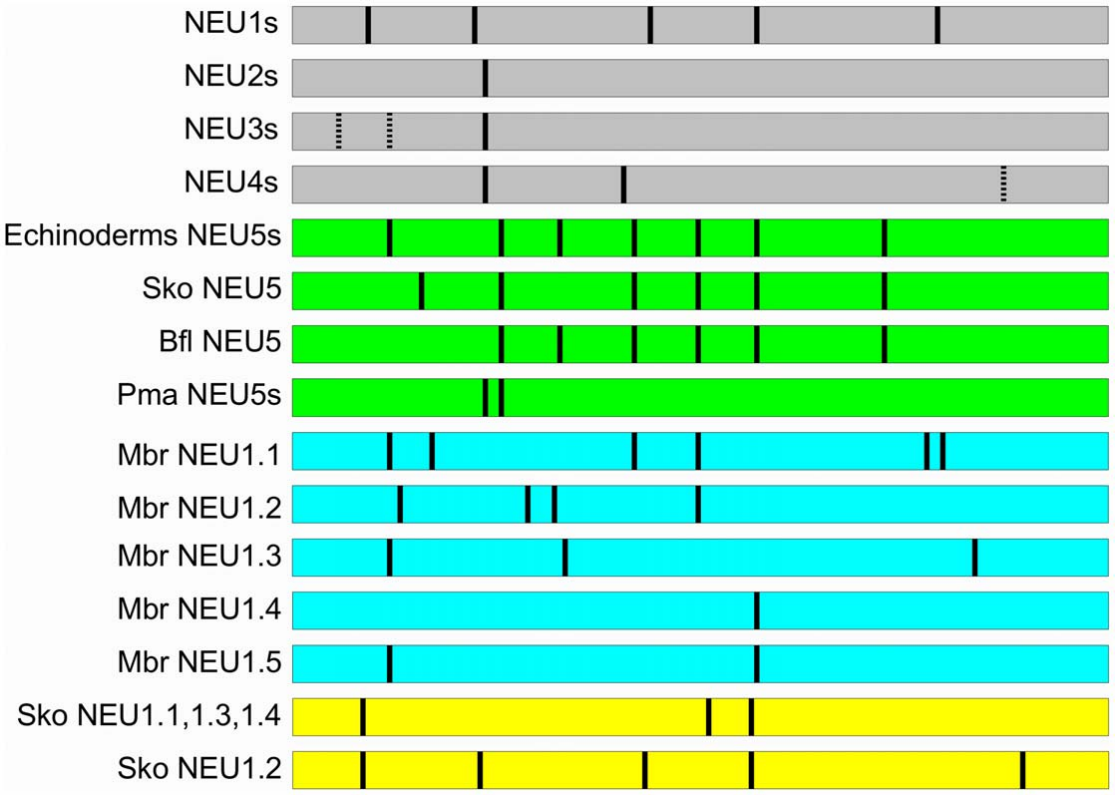
Exon structure analysis. Exon structure of the four already known sialidase subgroups (NEU1–4; grey bars), of the newly identified NEU5 sialidases (green bars), of the 5 sialidases from *M. brevicollis* (light blue bars) and of the NEU1 sialidases from hemichordate *S. kowalevskii* (yellow bars). Horizontal bars represent the protein sequence and the position of the exon junctions within the polypeptides are indicated by the black lines. Dotted black lines in NEU3 subgroup indicate additional exons found only in the avian species analyzed (*G. gallus, T. guttata*), while in NEU4 indicate an additional exon find only in *X. tropicalis*. The bar representation of the protein sequences is not in scale with the exon length, but the reciprocal position of exon junctions in different subgroups are respected.

Using the procedure described in Material and Methods we were able to determine the exon structure for the protein coding sequence (CDS) of all the newly identified sialidase genes, except those from *S. vortens, K. brevis, O. lobularis* and the sea urchin *P. lividus,* due to the lack of genome sequence, and that of NEU4 from *E. caballus,* due to the presence of a sequence gap. Most of the genes showed a conserved exon structure resembling those of mammalian sialidases: 6 exons for NEU1, 2 exons for NEU2 and NEU3 and 3 exons for NEU4. An exception is represented by NEU3 and NEU4 sequences from avian species that posses an additional exon compared to the classical subgroup organization. The five NEU1 sequences from the marine choanoflagellate *M. brevicollis* showed a variety of organizations: NEU1.1 with 7 exons, NEU1.2 with 5 exons, NEU1.3 with 4, NEU1.4 with 2 and NEU1.5 with 3. Peculiar exon organizations (4 exons) were also found in NEU1.1, NEU1.3 and NEU1.4 from the hemichordate *S. kowalewskii,* while NEU1.2 resembles the canonical exon structure found in mammalian NEU1s. The NEU5 subgroup revealed an heterogeneous state, with members having slightly different exon organizations which is overall dissimilar from that of other sialidases. A notable exception is represented by the NEU5s from the lamprey *P. marinus* that revealed an exon structure similar to the one observed in NEU3 and NEU2. All the data are represented in Figure 6.

### Phosphorylation Analysis Identified Globally Conserved Phosphorylatable Sites as well as Sites Conserved Only in Specific Subgroups or Evolutionary Classes

**Figure 7:**
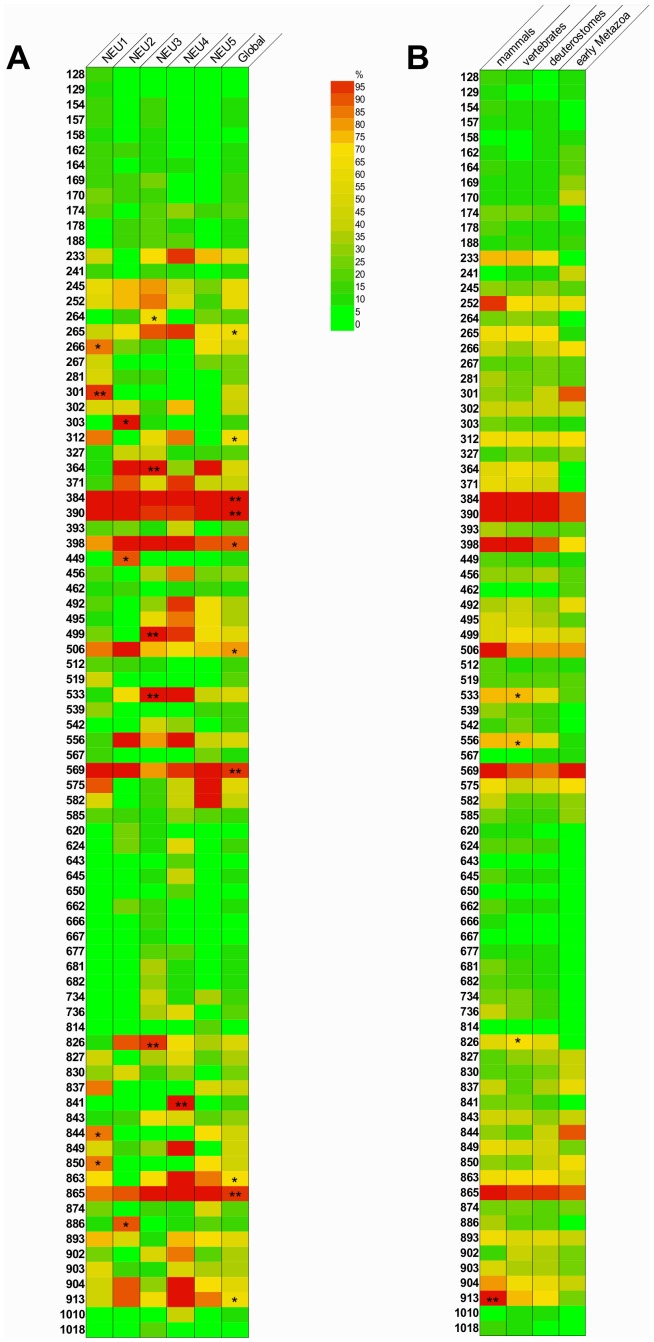
Conservation of phosphorylatable residues. Heatmap representation of the level of conservation of the 84 predicted phosphorylation sites is given for the global set of 83 sialidases and for each one of the five sialidase subgroup in (A) and for the 4 taxonomical groups in (B). Phosphorilatyon sites are identifeid according to their absolute position in the global alignment of the 83 sialidase sequences. Globally (A) and specific subgroup (B) sites showing statistical significant conservation (p<0.01) are indicated: * corresponds to sequence conservation ≥60%; ** corresponds to conservation ≥90%.

Conservation of the predicted phosphorylation sites in each one of the five sialidase subgroups (NEU1–5) were analyzed as described in Materials and Methods. Netphos 2.0 prediction identified 27 sites in NEU1, 13 in NEU2, 28 in NEU3, 31 in NEU4 and 17 in NEU5. Considering that some of these sites represent an equivalent position in the multiple alignment, the overall dataset resulted in 84 distinct putative phosphorylated positions. Among sites with a significant conservation in the global alignment, we identified 6 sites showing more than 60% overall conservation and 4 sites showing more than 90% overall conservation. Interestingly, we also identified 13 sites that are significantly conserved only in specific subgroups with a level of conservation ≥60% (conservation of each phosphorylated position in the NEU1–5 subgroups is represented in Figure 7A). We also considered the conservation of the predicted phosphorylation sites throughout evolutionary groups (as described in Materials and Methods) and found 4 positions that are significantly conserved only in one specific class of organisms (see Figure 7B). The 27 most interesting residues are reported in Table 4 and the complete list of the phosphorylation positions is reported in Tables S2 and S3.

**Table 4:** Conservation of phosphorylatable sites.

NetPhos prediction	Absolute position	NEU1	NEU2	NEU3	NEU4	NEU5	Total
NEU3 S79	264	0	12.5	63.15*	0	25	18.07
NEU2 S43; NEU3 T80; NEU4 S57	265	35.13	62.5	89.47	90.91	62.5	60.24*
NEU1 S100	266	83.78*	25	10.52	0	62.5	48.2
NEU1 S114	301	94.59*	0	0	0	0	42.17
NEU2 Y59	303	0	100*	5.26	0	0	10.84
NEU4 S77	312	81.08	0	57.89	81.81	0	60.24*
NEU3 T145	364	5.4	100	100*	27.27	100	48.19
NEU1 S174	384	97.30	100	100	100	100	98.80*
NEU1 S180; NEU3 S170; NEU4 S147	390	97.30	100	94.74	90.91	100	96.39*
NEU1 S188; NEU3 T178; NEU5 T136	398	75.67	100	100	100	87.5	87.95*
NEU2 S173	449	2.70	87.50*	0	0	0	9.64
NEU5 Y196	499	21.62	0	100*	90.91	62.5	50.6
NEU1 S239	506	83.78	100	73.68	63.63	75	79.51*
NEU5 S219	533	5.4	62.5	100*	100	37.5	48.19
NEU1 S295; NEU2 S248; NEU3 S289;NEU4 S265; NEU5 S254	569	100	100	78.94	90.91	100	93.4*
NEU3 T368	826	6.25	87.5	94.73*	63.63	33.33	47.36
NEU4 S409	841	0	0	0	100*	0	14.47
NEU1 S349	844	84.37*	0	0	0	66.66	40.78
NEU1 S355	850	84.37*	0	0	0	66.66	40.78
NEU4 S429	863	68.75	0	68.42	100	83.33	67.1*
NEU1 Y370; NEU4 Y431; NEU5 Y353	865	84.38	87.50	100	100	100	92.11*
NEU2 S347	886	6.25	87.50*	0	9.09	16.67	14.47
NEU1 S410; NEU3 T436; NEU5 S388	913	40.62	87.5	63.15	100	83.33	63.15*
		**Mammals**		**Vertebrates**		**Deuterostomes**	
NEU5 S219	533	71.43		71.15*		51.35	
NEU2 S238; NEU4 S255	556	71.43		71.15*		54.05	
NEU3 T368	826	52.38		67.31*		50.72	
NEU1 S410; NEU3 T436; NEU5 S388	913	100*		75		65.22	

Percentage of conservation for each phosphorylatable site across the specified sialidase subgroup and in the total dataset of 83 sequences are reported. Positions indicated refer to the absolute positions of the predicted phosphorylatable site in the global multiple alignments comprising all the 83 sialidase sequences (see Multiple FASTA alignment S1). Percentage of conservation for each phosphorylatable site are reported in the second part of the table for mammals, vertebrates and deuterostomes. Statistically significant (p<0.01) conservation values are marked with * when they exceed 60% conservation.

### Phylogenetic Analysis Reconstructed the Evolution of Sialidase Protein Family

**Figure 8:**
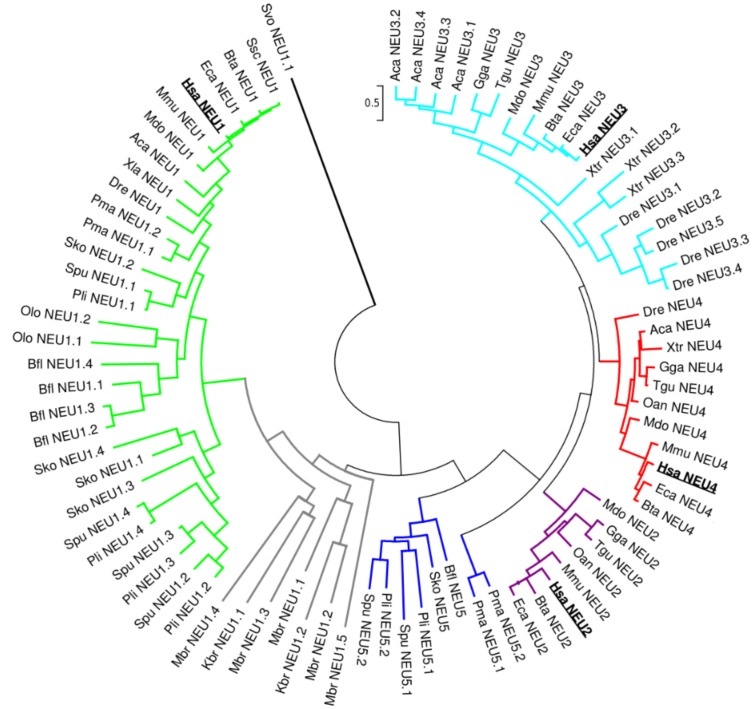
Complete phylogenetic tree of the sialidase gene family. Complete phylogenetic tree of the sialidase gene family reconstructed using Maximum Likelihood method and WAG+G amino acid substitution model. The tree is drawn to scale, with branch lengths measured as number of substitutions per site. The tree with the highest log likelihood (-38941.9521) is shown. Human sialidases are reported in bold. Different subgroups are represented with different colors: NEU1 in green, NEU2 in purple, NEU3 in cyan, NEU4 in red and NEU5 in blue. Branches containing the protist *K. brevis* and the Choanoflagellata *M. brevicollis* are represented with grey lines. Bootstrap analysis was conducted with 1000 replicates (bootstrap values are reported in detailed trees in Figure 9). Sialidase from the flagellate *S. vortens* (Svo NEU1) is placed at the tree root. The tree was constructed, visualized and manipulated using MEGA5.

**Figure 9:**
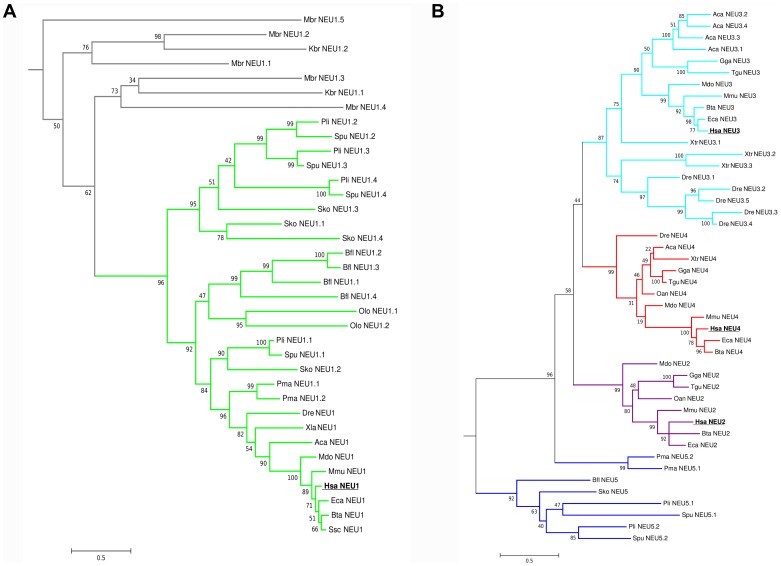
NEU1 and NEU2–5 phylogenetic subtrees in sialidase protein family. Detailed view of the phylogenetic subtrees of NEU1 sialidase subgroup (A) and of the other subgroups (B). The newly identified NEU5 subgroup emerges at the root of the NEU2, 3 and 4 subclasses. Bootstrap values are indicated for every node. Color code is used for different subclasses: NEU from protist *K. brevis* and from choanoflagellate *M. brevicollis* in grey, NEU1 in green, NEU2 in purple, NEU3 in cyan, NEU4 in red, NEU5 in blue. The tree is drawn to scale, with branch lengths measured in the number of substitutions per site.

The complete phylogenetic tree for the entire sialidase family is shown in Figure 8. Details of the subtree reconstructed for the NEU1 subgroup and for the other 4 subgroups are shown separately in Figure 9.

NEU1 subgroup is isolated in a separate branch of the complete sialidase tree and NEU1-like sequences are present in all the considered organisms. NEU1 putative ortholog identified in protist *S. vortens* emerges at the root of the tree. Sequences from the Metazoa closest relative, *M. brevicollis,* are located at low levels in the NEU1 subgroups branch together with a sialidase from the other protist considered (*K. brevis)*. The newly identified NEU5 subgroup is located at the root of the tree containing the membrane-associated forms NEU3 and NEU4 and the cytosolic sialidase NEU2. Sequences belonging to the NEU5 group were identified in early Deuterostomia (*S. purpuratus* and *P. lividus*) and in precursors of Chordata (*B. floridae*) and Vertebrata (*P. marinus*).

Interestingly, NEU2 subgroup was identified only in avian species and mammals and emerges as a branch parallel to NEU3 and NEU4 subgroups, even if NEU2 sequences have a slightly higher similarity to NEU4 subgroup.

Multiple putative co-orthologs were detected in our study in various species for NEU1 and NEU3 subgroups. Several copies of NEU1-like genes are present particularly in ancient organisms, from *M. brevicollis* to the chordate ancestor *B. floridae*, while tandem duplications of NEU3 genes have been identified in teleosts (*D. rerio*, as already reported in [16]), amphibians (*X. tropicalis*) and reptiles (*A. carolinensis*). Also the NEU5 subgroup showed duplicated genes in both Echinodermata (*S. purpuratus* and *P. lividus*) and Agnata taxon (*P. marinus*).

### NEU1–4 Sialidase Genes Show Conserved Synteny Throughout Vertebrata

Overall a high level of synteny was observed for *NEU1–4* genes using Genomicus in Vertebrata (Figure S2), while genomic data from lower organisms are often available as short contigs, thus hampering the evaluation of synteny.

### The Sialidase Family Shows Co-evolution with Sialyltransferases

**Figure 10:**
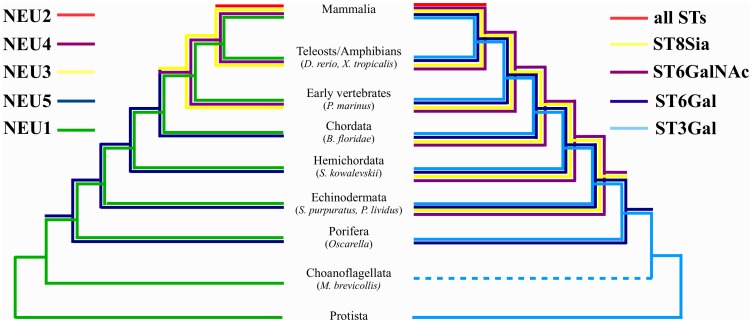
Co-evolution of the sialidase (NEU) and sialyltransferase (ST) gene families reconstructed using Mesquite software. Each subgroups in the two families is represented by a line of different color, NEUs on the left side, STs on the right side. The development of new classes of glycoconjugates in Echinodermata corresponds to the evolution of the NEU5 enzyme in sialidases. The appearance of the complete set of STs in Mammalia corresponds to the appearance of NEU2 in sialidase family. ST line pointing to Choanoflagellata is dotted since we were unable to retrieve any STs related sequence in Choanoflagellata and thus the actual presence of STs in this taxon can not be confirmed. Sialyltransferases are named according to [57].

Co-evolution of sialidase and sialyltransferase (ST) enzymes was evaluated as described in Material and Methods and shown in Figure 10. Both enzyme families are present from the Protista and show increase in complexity starting from echinoderms, with the appearance of sialidase NEU5 and STs ST6GalNAc and ST8Sia. The establishment of the complete set of STs subclasses in mammals corresponds with the appearance of NEU2 in the sialidase family.

### Comparison of Structural Models Shows Interesting Differences in Residues Interacting with the Substrate as well as Insertions in the Loops Connecting the β-strands of the Propeller

Structural models based on NEU2 template were predicted with I-Tasser for human sialidases and for NEU1 from *M. brevicollis*, *O. lobularis*, *S. purpuratus*, *B. floridae* and *P. marinus* and the newly identified NEU5 from *S. purpuratus*, *B. floridae* and *P. marinus*. All models fit well with the β-barrel structure typical of sialidases [31]. After superimposition on NEU2, the architecture of the catalytic site resulted highly conserved, with the 6 key catalytic residues showing an almost perfect correspondence in all evaluated models (Figure 1A). The positions of the other 5 residues that in NEU2 interact with the competitive inhibitor DANA (in blue in Figure 1B) show more flexibility resulting in different organizations of this portion of the catalytic crevice in the sialidase NEU1 of the organisms considered in this study. Moreover, in NEU2 3D structure, as well as in NEU3, NEU4 and NEU5 predicted models, E111 residue is located on a flexible loop containing a short α-helix, which is absent in proteins from the NEU1 subgroup (Figure 4C). Finally, the comparison of known structures from viruses (such as *Newcastle virus, Influenza B* and *Influenza N9* viruses), bacteria (such as *C. perfrigens, S. pneumoniae, V. cholerae, S. tiphimurium*) and trypanosomes (such as *Trypanosoma cruzi*) with NEU2 of human origin revealed the presence in the former ones of complex additional domains, such as lectin domains [32], [33], located in the loops connecting the various β-sheet of the sialidase structure (Figure 2).

## Discussion

### Primary Structure Analysis

The analysis of the primary structure of the sialidases considered in this study using subfamily logo allowed us to identify several residues that are characteristic of the single sialidase subclasses. Since several of them are likely exposed on the enzyme surface they may be involved in protein-protein interactions or may play a role in cellular localization of the enzyme. A complete list of these differences is reported in Table 3. Identification of these residues can guide future functional studies and thus help in understanding peculiar aspects of sialidase biology. Analysis of the known lysosomal localization signals present in NEU1s [30] revealed that both the N-terminal peptide and the C-terminal sequence signal Y(-4) are conserved only in higher organisms. The first seems conserved only in mammals (Figure 5A), while the aromatic residue seems retained trough the Chordata phylum (Figure 5B). These results support the idea that the recruitment of NEU1 in the lysosome largely relies on its interaction with PPCA and β-Gal within a trimeric protein complex [34]. Indeed, residues essential in NEU1-PPCA interaction [29] are selectively conserved in NEU1 while they are completely lost in other sialidase classes, even in the ancestral NEU5 (Figure 5C). In this perspective, mutations of these residues could have been a key factor in the appearance of the new enzyme subtypes allowing for sub-cellular localizations other than lysosomes.

We also evaluated the presence and conservation of phosphorylation sites in the five sialidase subgroups as well as in four main evolutionary groups (early metazoan, deuterostomes, vertebrates and mammals). Interestingly we have found 4 putative phosphorylation sites (at positions 384, 390, 569 and 865 in the multiple alignment) that are >90% conserved in all sialidases considered, suggesting these may represent real post-translational modification spots. We were also able to identify sites conserved only in specific subgroups (Figure 7A): residue at position 266, 301, 844 and 850 are conserved only in NEU1, at positions 303, 449 and 886 only in NEU2, at positions 264 and 826 only in NEU3, at positions 841 only in NEU4, at position 499 and 533 both in NEU3 and NEU4. These sites may reflect differences in enzyme modifications that can play a role in determining sub-cellular localization and/or regulating enzyme activity. Considering these data in an evolutionary classification of sequences also revealed 4 sites that are conserved only in specific taxonomic groups (Figure 7B). Residues at position 913, specifically conserved only in mammals, and at position 533, 556 and 826 conserved only in vertebrates are of particular interest. They may represent differences in phosphorylation-based regulatory mechanisms that may contribute to different behavior of the sialidase enzymes in different organisms, related to the role(s) played by these enzymes throughout evolution. Indeed, phosphorylation is known to play a role in mammals in modulating the localization and enzyme activity of sialidases [35], particularly NEU3 [36], [37], and it is reported also on trans-sialidase from *T. cruzii* [38]. Overall, our data provide a valuable resource to identify important residues for future experimental studies on phosphorylation mechanisms acting on sialidase enzymes.

In addition, for the organisms with a complete genomic sequence available, we also determined the exon structure of the CDS (Figure 6) to further support our subgroup assignment and to highlight evolutionary changes. The five NEU1 sequences from *M. brevicollis* show a peculiar organization ranging from 2 exons (Mbr NEU1.4) to 7 exons (Mbr NEU1.1), with no similarity with the classic organization of NEUs from higher organisms. Although this may reflect an actual difference between the ancestral genes and the modern form of NEU1, we can not exclude the presence of artifacts, being the genome sequence of this organism still in an early unfinished state. The lack of a publicly available genome sequence for protists and for the sponge *O. lobularis* prevented us to evaluate the state of NEU1 orthologs out of Deuterostomia. In this latter taxon, with the exception of sequences from the hemichordate *S. kowalevskii*, the NEU1 subgroup is quite uniform, showing the typical 6 exon structure of mammalian NEU1. Similarly, all NEU3 and NEU4 orthologs from vertebrates share the same exon structures, but the sequences from avian species, such as *G. gallu*s and *T. guttata,* that revealed a peculiar exon organization. This may be due to specific events occurred in an ancestor of modern birds.

### Phylogenetic Analysis of Sialidases

Our survey of the genomic and protein databases allowed the reconstruction of the state of sialidase enzymes in the Eukarya domain of life, evidencing a wide distribution of this class of enzymes from proto-eukaryota (such the protist *S. vortens*) to higher mammals. Results from our phylogenetic analysis are in agreement with the most accredited hypothesis that places the origin of sialidase/neuraminidase enzymes in bacteria, followed by the development of specialized forms in higher organisms [2], [6]. The lack of complete genomic data in ancient organisms limited our ability to reconstruct a complete CDS in some species giving only partial protein sequences. This may generate unexpected results in multiple alignments process and phylogenetic tree inference, resulting in low bootstrap values for particular tree nodes.

Interestingly, we found frequent duplications affecting genes encoding NEU1 enzymes, particularly in organisms more ancient than vertebrates. However, the lack of EST or RNA-Seq sequence data makes it impossible to establish if all these multiple enzymes are expressed in the same organisms or if they represent pseudogenes.

It is intriguing to hypothesize that these multiple gene copies have played a role in the development of the other members of the sialidase family by a classical mechanism of subfunctionalization. Analysis of the phylogenetic tree reconstructed for NEU1 and coalescence analysis allowed us to develop a putative model to explain the state of this enzyme in the organisms considered in the present study. First of all, NEU1 sequences from *M. brevicollis* and *K. brevis* (Dinofalgellate) resulted in a separate branch at the root of NEU1 subgroup (in grey in Figure 8), suggesting that the enzyme identified in these early Eukaryotes is an ancient precursor of the present sialidases. Based on the 5 *NEU1* paralogs identified in *M. brevicollis* and the state of sialidase orthologs in Protista, we speculate the existence of an ancestor of Metazoa with at least 3 copies of *NEU1* gene. Three copies of the gene have been inherited by descendants with one species-specific duplication occurred in the Echinodermata ancestor, leading to 4 copies of *NEU1* in *S. purpuratus* and *P. lividus*, and in Hemichordata, leading also to 4 copies in *S. kowalevskii*. Two duplications have probably occurred in *B. floridae* (Chordata ancestor) since this organism shows the presence of 5 copies of the *NEU1* gene. Loss of redundant gene copies has finally occurred in Vertebrata, with *P. marinus* (an ancestral vertebrate) showing only 2 copies and higher vertebrates possessing a single copy of *NEU1* gene.

Also *NEU3* gene revealed the occurrence of duplication events leading to multiple copies of the gene in *D. rerio*, *X. tropicalis* and *A. carolinensis*. In these cases the genes are duplicated in tandem forming a cluster of multiple *NEU3s*. These organisms have probably inherited this cluster from a common ancestor. We have evidences from EST data that four out of five copies are expressed in *D. rerio* (*Neu3.4* is the only gene with no EST support), two out of three in *X. tropicalis* (*Xtr_Neu3.2* is supported only by a single partial EST sequence) and one out of four in *A. carolinensis* (*Aca_Neu3.1* is the only one with EST support). This suggests an ancient duplication event followed by a progressive loss of the redundant copies.

Besides the orthologous genes encoding the 4 known members of the sialidase gene family, our phylogenetic analysis allowed the identification of a new form of the enzyme, named NEU5, showing comparable level of sequence similarity with both NEU3 and NEU4 (Table 2). This new class of sialidase has been identified in Echinodermata (*P. lividus* and *S. purpuratus*), Hemichordata (*S. kowalevskii)*, Cephalochordata (*B. floridae)* and Agnatha vertebrates (*P. marinus)*. It is interesting that the appearance of a class of sialidase enzyme other than NEU1 occurred first in echinoderms, at the same time of the dramatic increase in sialic acid diversity [6]. These organisms are also the first example of an intensive use of modified sialic acids on glycoconjugates on the cell surface and in cell-to-cell interactions. The pattern of sialoglycoconjugates on the gamete surface is in fact essentials in echinoderms, which diffuse gametes directly in the water, in order to avoid cross-species fertilization and the generation of sterile or non-vital embryos [39], [40]. The estimates of evolutionary divergence between sialidase subgroups (see Table 2) support an equal similarity of NEU5 proteins to both NEU3 and NEU4. In addition, considering the sequence similarity of NEU5 with mammalian NEU3 and NEU4, which are well known membrane associated enzymes [1], we speculate that the newly identified NEU5 also could be a membrane-associated enzyme. The peculiar exon structure of most NEU5 genes (Figure 6) is significantly different from those of other NEU subgroups, supporting the idea that this enzyme represents a distinct form of sialidase and not simply an ancestral NEU2, NEU3 or NEU4. The class of membrane-associated enzymes, namely NEU3 and NEU4, may have evolved by a process of progressive differentiation from NEU1, resulting in NEU5 as an intermediate state. The position of NEU5s from *P. marinus* (an early vertebrate) in a single branch at the root of the sub-tree containing NEU3, NEU4 and NEU2, separated from other NEU5 sequences, suggests that these proteins are closer to the modern membrane bound forms of sialidases. Indeed, also the exon structure of *P. marinus* NEU5s resembles that of NEU3 more than those of NEU5 from other organisms or NEU1 (Figure 6), further supporting the hypothesis that NEU5s from lamprey represent an ancestor of the modern membrane-bound sialidases, NEU3 and NEU4, present in vertebrates.

Overall, NEU1 was confirmed as the ancestral sialidase enzyme detected in all the organisms analyzed and showing a significant sequence similarity, higher than the other members of the family, also with bacterial sialidases. These data further support the hypothesis that both sialic acids and sialidases have an ancient bacterial origin, related to the polysialic acid and bacterial wall biology, and they have then evolved as key molecules in cell-to-cell and cell-to-pathogens interactions generating the actual complex pattern of sialic acids and sialic acids-related enzymes [6], [9], [41].

Furthermore, our results demonstrate that sialidases NEU2, NEU3 and NEU4 evolved as distinct enzymes in vertebrates and they may represent a response to complexity in sialic acid modifications and increased use of sialic acid based interactions in cell-to-cell communication and immune system regulation. The NEU2 enzyme seems to be the last member of the sialidase family which has evolved, appearing only in Amniota. Indeed a NEU2 otholog has been identified in avian species (such as *G. gallus* and *T. guttata*) but it is absent in bony fishes (*D. rerio)* and amphibians (*X. tropicalis)*. The fact that we were not able to identify any NEU2 related sequence in *A. carolinensis*, a lizard belonging to the Sauria clade and thus near to avian species, could be explained by a selective loss of NEU2 enzyme in this organism. Indeed, DNA loss events are particularly frequent in the *A. carolinensis* genome, in part as a consequence of the high activity of transposable elements in this species [42], [43]. However it is also possible that NEU2 ortholog in lizard is located in a gap in the genomic assembly of this organism making it impossible to retrieve it by our sequence database searches. During the revision of this paper, the genome sequence of *Chrysemys picta bellii* (western painted turtle) a member of the Sauropsida, has been made available. Using the same reciprocal best BLAST hits approach described in Material and Methods, with the human NEU2 protein as query, we retrieved a putative NEU2 ortholog, showing 55% sequence identity with the human protein. This data indicates the hypothesis that NEU2 is present in Sauropsida taxon and that the possible lack of NEU2 sequences in *A. carolinensis* is peculiar of this organism.

Taken together our data on the sialidase gene family evolution are compatible with results obtained in survey of the sialyltranferase (ST) enzymes [13], [14]. Comparison of the phylogenetic trees for the two families revealed that the development of new forms of STs, resulting in new kinds of sialoconjugates, are evolutionary concomitant with the appearance of a new class of sialidase enzymes (Figure 10) tailored to degrade the new molecule(s) or modulating its(their) action.

We have extensively searched public databases for sialidase related sequences in Protostomia, with particular attention to those in which sialic acid or STs enzymes has been reported, such as Cephalopoda (common squid) [44] or insects (fruit fly) [45]. We were unable to find any significant match with the query sequences even in those organisms with good genomic and transcribed sequence coverage, such as the fruit fly, *D. melanogaster*. This is surprising considering that presence of sialic acids has been demonstrated in these organisms, even if limited to specific developmental stages or tissues, and that, at least in the case of *Drosophila*, there are also clear evidences of a complete sialoconjugates synthetic pathway [45]. The absence of sialidase enzymes in Protostomia can be explained by a generic lack of Asp-box containing hydrolases in this systematic group, as demonstrated by a recent survey conducted on this class of enzymes [46]. Moreover, studies on cultured insect cells have revealed that, even if these cells lack one or more enzymes essential for sialoconjugates synthesis, they show the presence of some simple species of this class of molecules, suggesting that Protostomia can in some way recycle sialic acids from the environment [47], [48]. However, sialic acids in Protostomia are always detected at low levels compared with Deuterostomia [6] and thus the action of a generic (glyco-)hydrolase, even if with low efficiency on sialic acid containing substrates, may be sufficient in these organisms and substitute the specific activity of sialidases. Studies on *Penaeus japonicus* (a crustacean, also known as Kuruma shrimp) [49] and *Triatoma infestans* (a blood-sucker insect belonging to the Hemiptera order, known as barber bug) [50], [51] have also revealed the presence of two secreted proteins with a molecular weight of about 32 and 26 kDa, respectively, acting as sialidase. Because all sialidases studied so far, as well as the proteins identified in this work, have always a molecular mass greater than 40 kDa, it seems unlikely that these enzymes from Protostomia fold in the typical β-propeller sialidase structure of sialidase. It is intriguing to consider these proteins as a new class of enzymes with sialidase activity specific for Protostomia, that might have arisen by convergent evolution. Following this hypothesis and considering that several protostomes rely on interactions with deuterostomes during their life cycle, acting as pathogens or commensal, it is possible that sialic acid related enzymes have been initially lost in Protostomia and then this biological pathway has been re-established via convergent evolution as an adaptation to sialic acids present in their Deuterostomia hosts.

### Structural Features of Sialidases

The analysis of the multiple sequence alignments among the sialidase sequences identified shows, as expected, a full conservation of the 6 residues essentials for the catalytic process, namely R21, D46, E218, R237, R304, Y334 [1], [3]. Data from the structural models obtained using NEU2 crystal structure [25] as template confirmed that these residues are also positionally conserved along the evolutionary tree (Figure 1A, D). On the contrary, the other residues interacting with the competitive inhibitor DANA, as identified by the study on the human NEU2 [25], show peculiar differences between NEU1-like enzymes and the other sialidases (Figure 1C). The position of these residues in the catalytic crevice, as deduced from the structural models, place them in contact with the glycerol chain of DANA, corresponding to the site in which most of the sialic acid modifications occurs (Figure 1B). This peculiar configuration suggests that these residues may play a role in modulating the substrate specificity of the different class of sialidases. The analysis of the loops surrounding the active site (Figure 4) showed that three of them, near the glycerol chain (Loop 3, 4 and 6), are generally shorter in NEU1-like enzymes compared to NEU5 and NEU2–3–4 polypeptides. These loops may interact with the substrate(s) and therefore modulate the range of sialic acid substrates accepted by the enzyme. This can be of particular relevance in the case of Loop 3 containing also the E111 residue (Figure 4C). This residue is placed on a short α-helix and interacts with DANA in NEU2 crystal structure, stabilizing the loop on the top of the catalytic crevice when the enzyme is bound to the competitive inhibitor DANA [25]. This loop is considerably shorter and both the short α-helix and the E111 residue are absent in NEU1 enzymes, leaving a more space/access for substrate(s) inside the catalytic site. In this perspective, these structural features could play a role in the evolution of the different forms of sialidases, restricting the substrate specificity from NEU1 (a lysosomal enzyme acting on a variety of sialoconjugates) to other forms of sialidases (acting preferentially on restricted classes of sialylated molecules).

Another structural signature of sialidase enzymes is the presence of multiple Asp-box domains [1]. These are well conserved motifs (SxDxGxxW/F) usually localized on loops connecting β-sheets at the opposite side of the catalytic crevice. Their presence can facilitate the folding of the β-propeller and stabilize its structure through hydrophobic interactions [46]. Our analysis confirmed the presence of 5 possible Asp-box sites within the Eukarya sialidase structures, placed in the loops connecting the third and the fourth β-sheets of the blades I–V of the β-propeller (Figure 2). Interestingly only Asp-boxes 2 and 3 are fully conserved in all classes of sialidases. Asp-box 4 is also well conserved, particularly the S and F residues, while Asp-boxes 1 and 5 are more divergent, being conserved only in NEU1 enzymes. In all cases the terminal aromatic W/F residue is still highly conserved supporting the hypothesis that it is the key element for the proper folding of Asp-box motifs [52]. The fact that NEU1 enzymes show the higher number, four, of conserved Asp-box motifs, further supports the conservation of this motif from bacterial sialidases, which also have several fully conserved Asp-boxes.

Analysis of multiple alignments and comparison of structural models of sialidases from different classes revealed that the loops connecting the antiparallel β-sheets of the β-propeller and not containing essential residues are highly variable in sequence and dimension. Extending our analysis to include also sialidases with defined structures from bacteria (*Clostridium perfrigens*, *Streptococcus pneumoniae*, *Vibrio cholerae*, *Salmonella tiphimurium*) and viruses (*influenza* virus and *Newcastle* virus) revealed that these loops are preferred sites for insertions of large amino acid motifs, which in bacteria often act as lectin domains (Figure 2). These domains may have a role in recognition of specific glycosylated molecules on the host cell surface which represent preferred antigens for the pathogen. Dimension of connecting loops is particularly variable in NEU3 and NEU4 enzymes, where they can be also considerably longer than in other sialidase enzymes [17]. In contrast, the loops are extremely short (usually 2–3 residues and never more than 5) in NEU1, resembling the more compact structure of bacterial sialidases (besides the ones with loops containing lectin domains). The multiple alignments also revealed that one of these loops is hyper-variable in dimension in vertebrates (Figure 3), with a length ranging from 2–5 aa in NEU1s to 60–100 aa in NEU4s, with a maximum of 200 aa in NEU3 from *G. gallus*. Interestingly this loop corresponds to the 80 aa region identified in mouse and human NEU4s [53], [54] and considered so far a signature of this particular group of sialidases. Mutagenesis experiments conducted removing this region from human NEU4 (unpublished results) and chicken NEU3 [17] have apparently no detectable effects on the evaluated enzyme properties and its function remains unknown. Looking at its position within sialidase structure, it emerges between two subsequent β-sheets and it is flanked by two highly conserved domains containing aromatic residues (Figure 2), which can act by stabilizing the blade structure, allowing the presence of even a large insertion without impairing the core architecture of the enzyme. This concept is also supported by the detailed study conducted on this loop in *G. gallus* NEU3 enzyme [17]. Overall, data collected on loops connecting the β-strands of the barrel in the sialidase structure indicate that these regions are preferred for rapid divergence, since they are subjected to low or no selective pressure. Exploration of new structural variants in rapid diverging loops has been proposed as an alternative mechanism driving the evolution of protein families and facilitating the creation of new functional domains [55], [56]. The compact core structure of the sialidase β-propeller is stabilized by several interactions between adjacent β-sheets, suggesting that it is thus able to tolerate also large loops insertions without impairment on the propeller architecture. Based on these observations, it is intriguing to speculate that long rapidly evolving loops played a role in the development of different forms of sialidases, modifying interaction with substrates or other protein partners. This process may has occurred in evolution of sialidase family generating the specialized membrane-bound NEU3 and NEU4 and the soluble NEU2. Data from the detailed characterization of *G. gallus* NEU3 further supports this evolutionary hypothesis [17].

### Final Considerations

We have generated the first comprehensive picture of the evolutionary pattern followed by the sialidase family of glycohydrolases, providing new insights into the picture of sialic acid biology and the peculiar features of this protein family. Our results further support the effectiveness of the phylogenetic approach as a tool for the study of the biology of protein families.

## Supporting Information

Figure S1 **Schematic diagram of the evolutionary relationships among the 21 organisms considered for this study.** Divergence time in millions of years (mya), as reported by TimeTree (http://www.timetree.org), are given for every branches. Main evolutionary taxa are in bold. Branches lenght are not representative of the evolutionary distance between species.
(PDF)

Figure S2 **Results from the analysis performed with Genomicus using the four human sialidases as query.** Genomicus reports the genomic organization of the region surrounding the gene of interest in various species and groups of Vertebrata. The gene of interest is located at the center of the block colored in light green.
(PDF)

Multiple FASTA Alignment S1 (FASTA)

Subgroup Alignments S1 **Multiple alignments of NEU1–4 protein sequences subgroups identified.** Alignment is elaborated with TexShade and shown in fingerprint style. The regions corresponding to the 6 blades that compose the sialidase β- propeller structure are indicated below the alignment.
(PDF)

Table S1 **Information on the 87 sialidase sequences.**
(PDF)

Table S2 **Conservation of the predicted phosphorylatable sites in each sialidase subgroups.**
(PDF)

Table S3 **Conservation of the predicted phosphorylatable sites in mammals, vertebrates and deuterostomes.**
(PDF)
