# Supplementary material for: New Insights on the Sialidase Protein Family Revealed by a Phylogenetic Analysis in Metazoa
Source: PLoS One. 2012 Aug 30;7(8):e44193. doi: 10.1371/journal.pone.0044193 (PMC3431349; doi:10.1371/journal.pone.0044193)

Figure S1. Schematic diagram of the evolutionary relationships among the 21 organisms considered for this study. Divergence time in millions of years (mya), as reported by TimeTree (http://www.timetree.org), are given for every branches. Main evolutionary taxa are in bold. Branches lenght are not representative of the evolutionary distance between species.

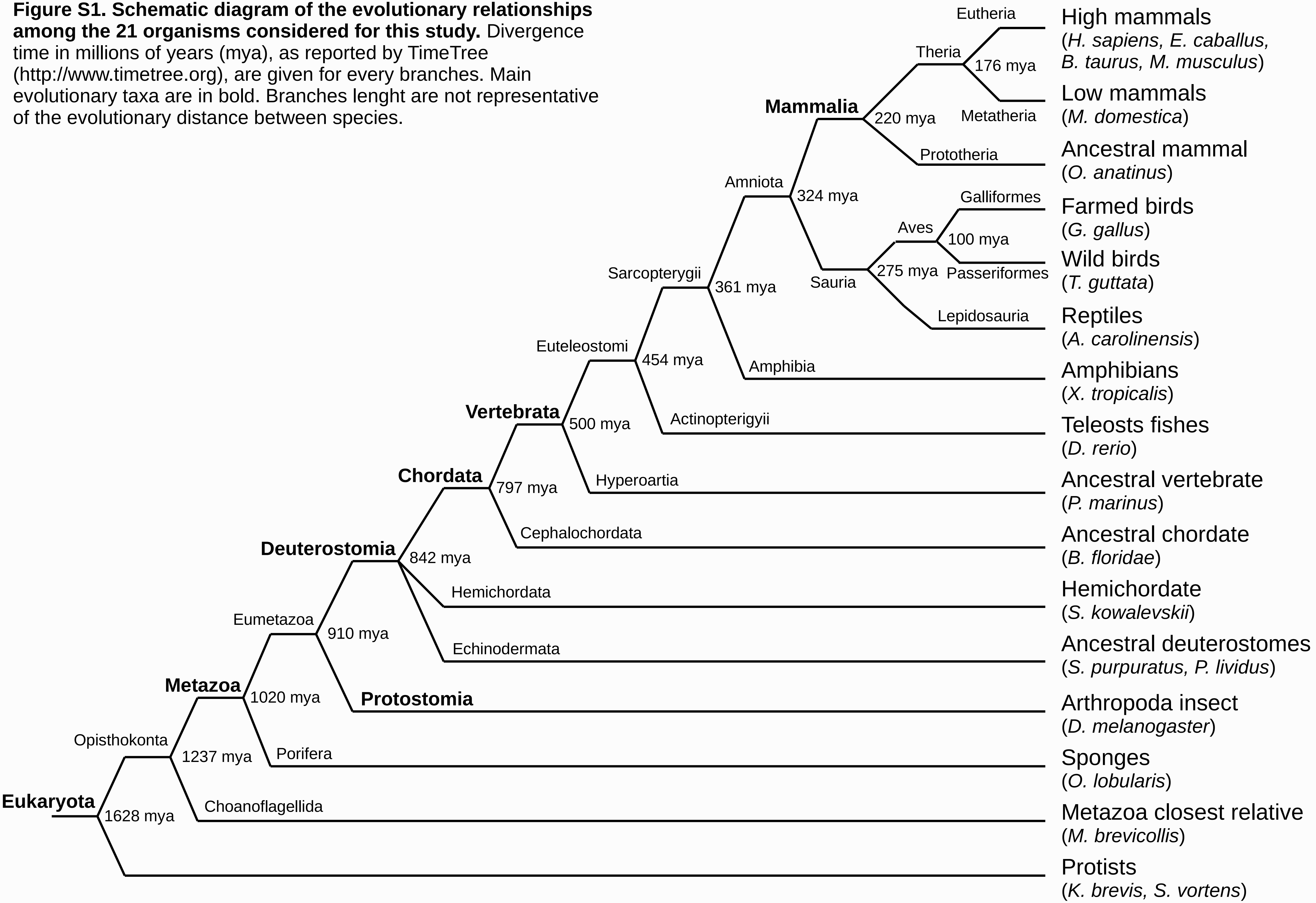

Supplement: Figure S1 — Schematic diagram of the evolutionary relationships among the 21 organisms considered for this study. Divergence time in millions of years (mya), as reported by TimeTree (http://www.timetree.org), are given for every branches. Main evolutionary taxa are in bold. Branches lenght are not representative of the evolutionary distance between species. (PDF) [file pone.0044193.s001.pdf]
