# Supplementary material for: New Insights on the Sialidase Protein Family Revealed by a Phylogenetic Analysis in Metazoa
Source: PLoS One. 2012 Aug 30;7(8):e44193. doi: 10.1371/journal.pone.0044193 (PMC3431349; doi:10.1371/journal.pone.0044193)
Supplement: Figure S2 — Results from the analysis performed with Genomicus using the four human sialidases as query. Genomicus reports the genomic organization of the region surrounding the gene of interest in various species and groups of Vertebrata. The gene of interest is located at the center of the block colored in light green. (PDF) [file pone.0044193.s002.pdf]

Figure S2. Results from the analysis performed with Genomicus using the four human sialidases as query. Genomicus reports the genomic organization of the region surrounding the gene of interest in various species and groups of Vertebrata. The gene of interest is located at the center of the block colored in light green.

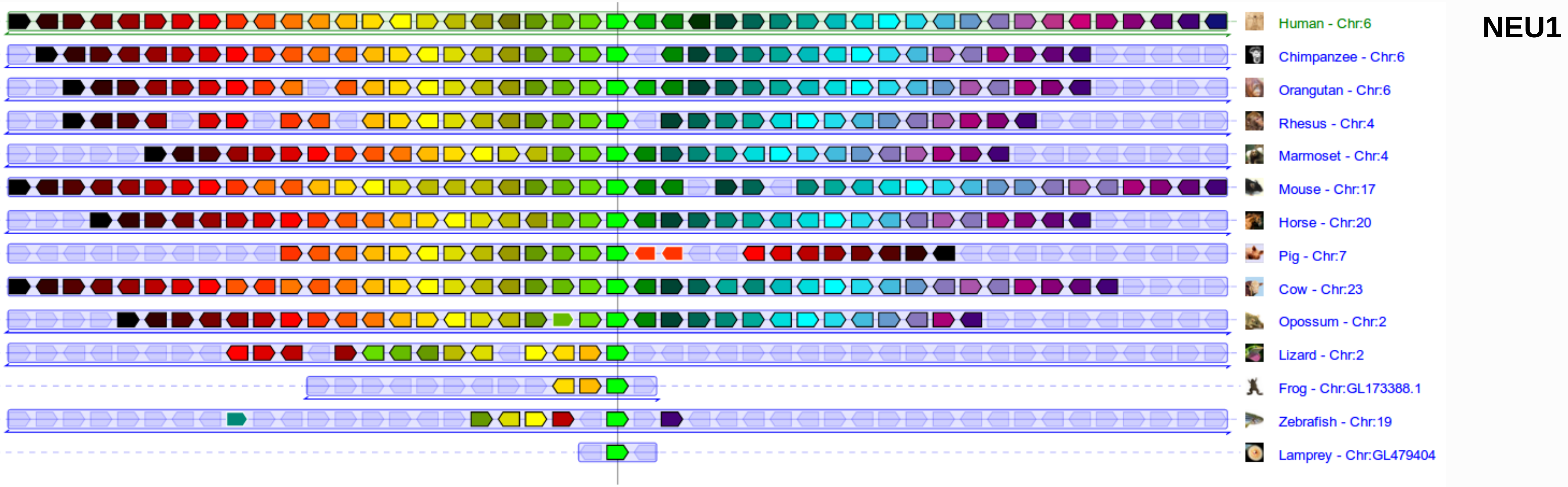

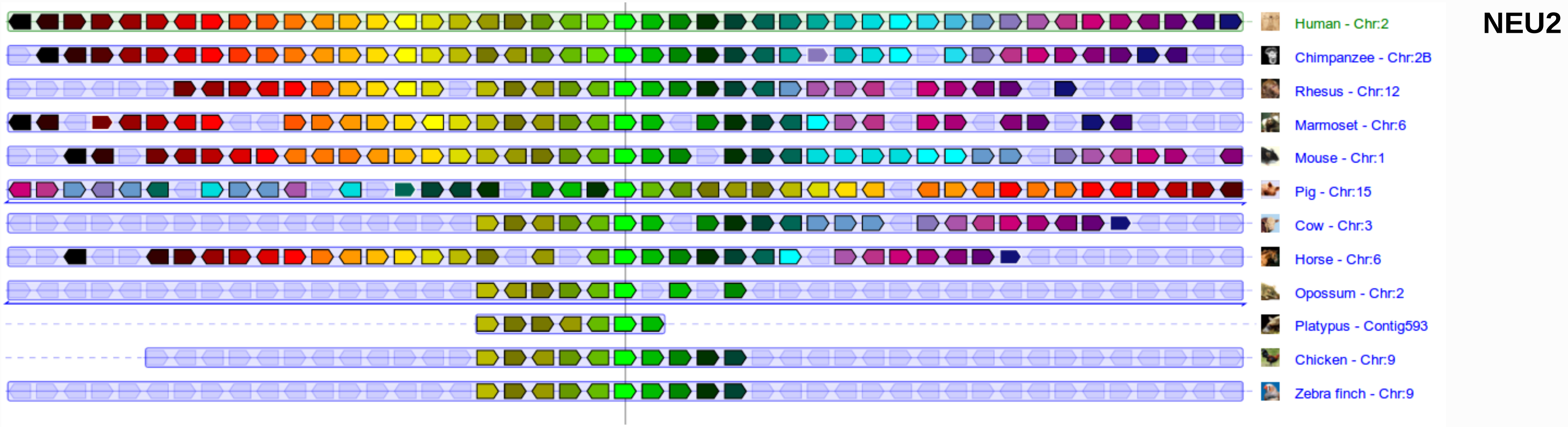

NEU3

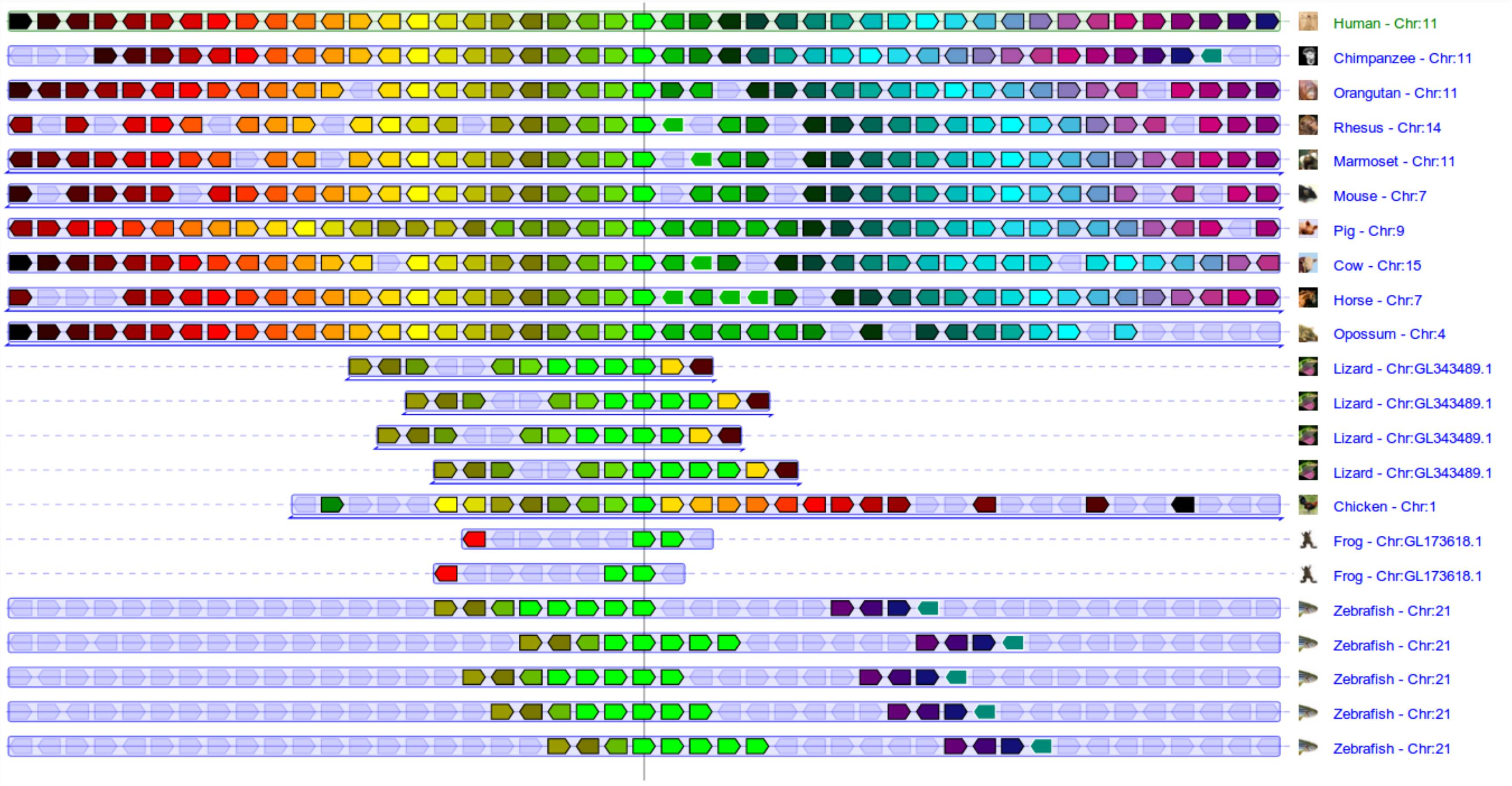
Human - Chr:11
Chimpanzee - Chr:11
Orangutan - Chr:11
Rhesus - Chr:14
Marmoset - Chr:11
Mouse - Chr:7
Pig - Chr:9
Cow - Chr:15
Horse - Chr:7
Opossum - Chr:4
Lizard - Chr:GL343489.1
Lizard - Chr:GL343489.1
Lizard - Chr:GL343489.1
Lizard - Chr:GL343489.1
Chicken - Chr:1
Frog - Chr:GL173618.1
Frog - Chr:GL173618.1
Zebrafish - Chr:21
Zebrafish - Chr:21
Zebrafish - Chr:21
Zebrafish - Chr:21
Zebrafish - Chr:21

NEU4

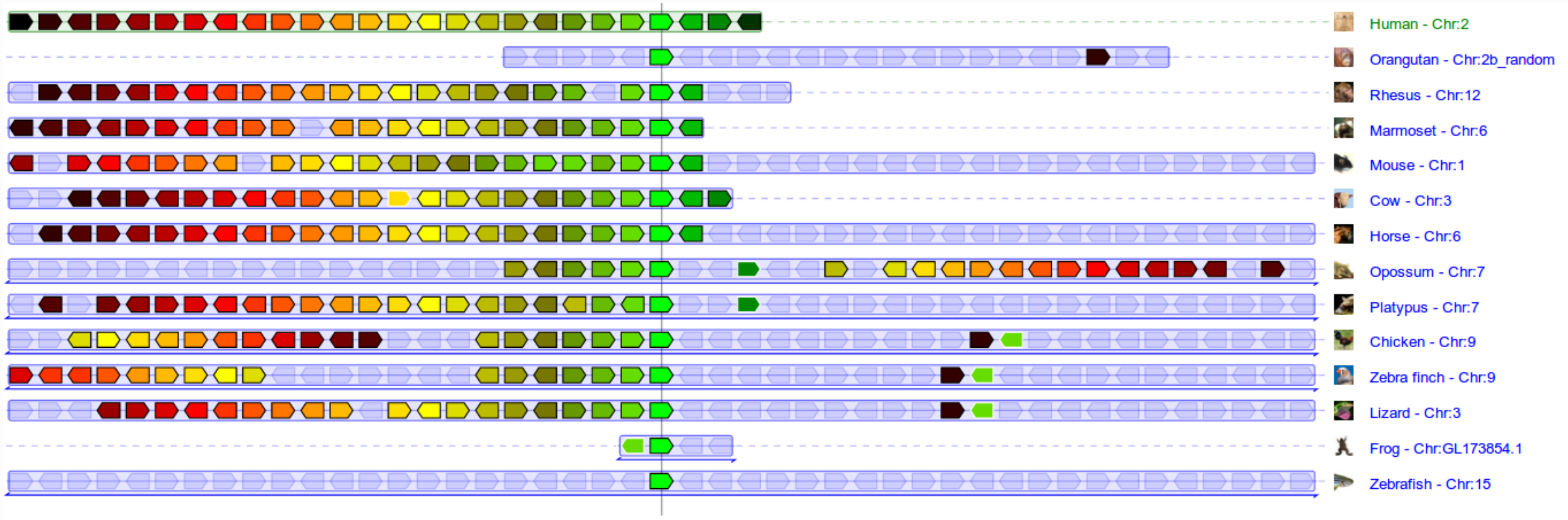
Human - Chr:2
Orangutan - Chr:2b_random
Rhesus - Chr:12
Marmoset - Chr:6
Mouse - Chr:1
Cow - Chr:3
Horse - Chr:6
Opossum - Chr:7
Platypus - Chr:7
Chicken - Chr:9
Zebra finch - Chr:9
Lizard - Chr:3
Frog - Chr:GL173854.1
Zebrafish - Chr:15
