# Supplementary material for: New Insights on the Sialidase Protein Family Revealed by a Phylogenetic Analysis in Metazoa
Source: PLoS One. 2012 Aug 30;7(8):e44193. doi: 10.1371/journal.pone.0044193 (PMC3431349; doi:10.1371/journal.pone.0044193)

NEU1 Subgroup Alignment. Multiple alignment of NEU1 protein sequences identified. Alignment is elaborated with TexShade and shown in fingerprint style. The regions corresponding to the 6 blades that compose the sialidase β-propeller structure are indicated below the alignment.

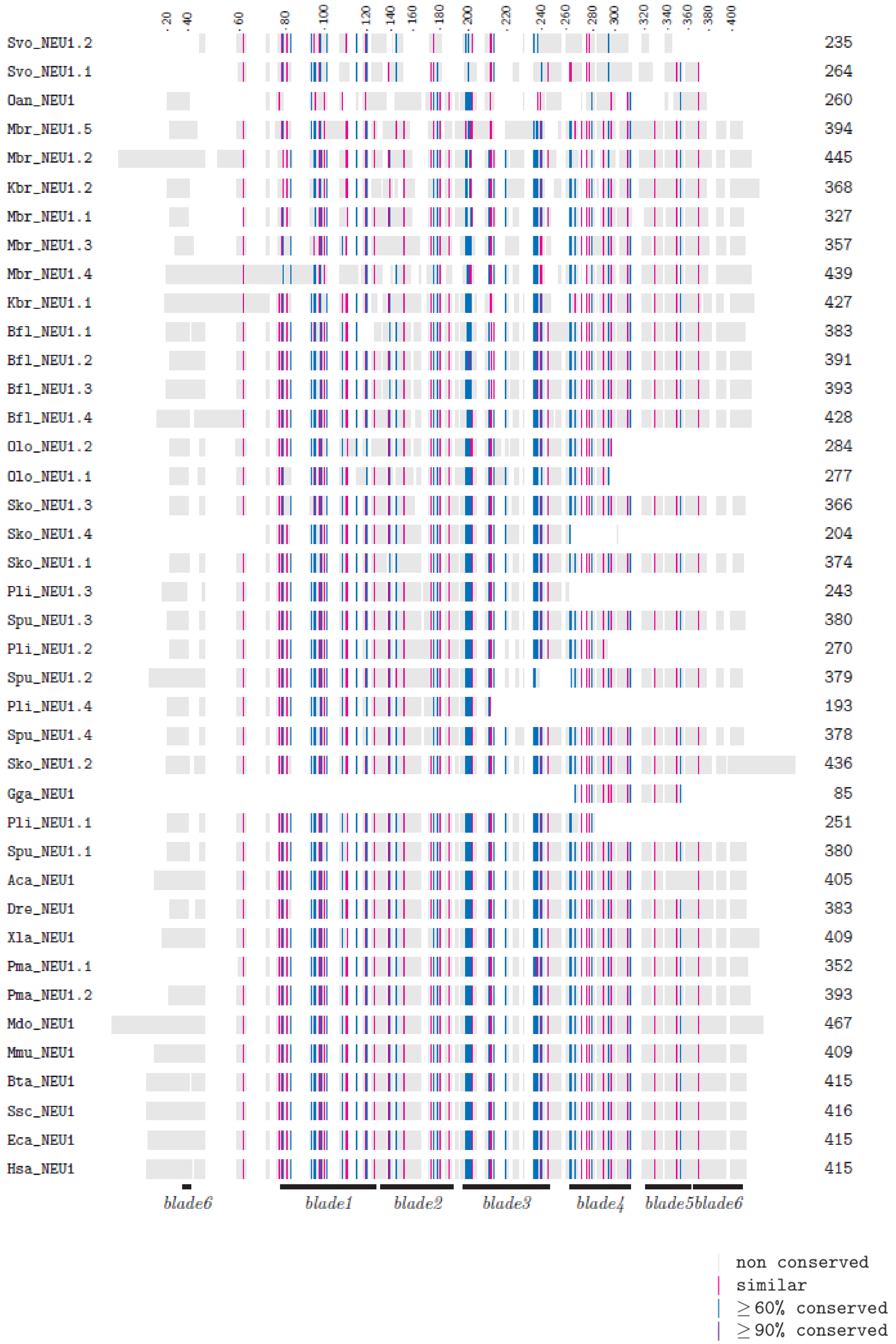

NEU2 Subgroup Alignment. Multiple alignment of NEU2 protein sequences identified. Alignment is elaborated with TexShade and shown in fingerprint style. The regions corresponding to the 6 blades that compose the sialidase β-propeller structure are indicated below the alignment.

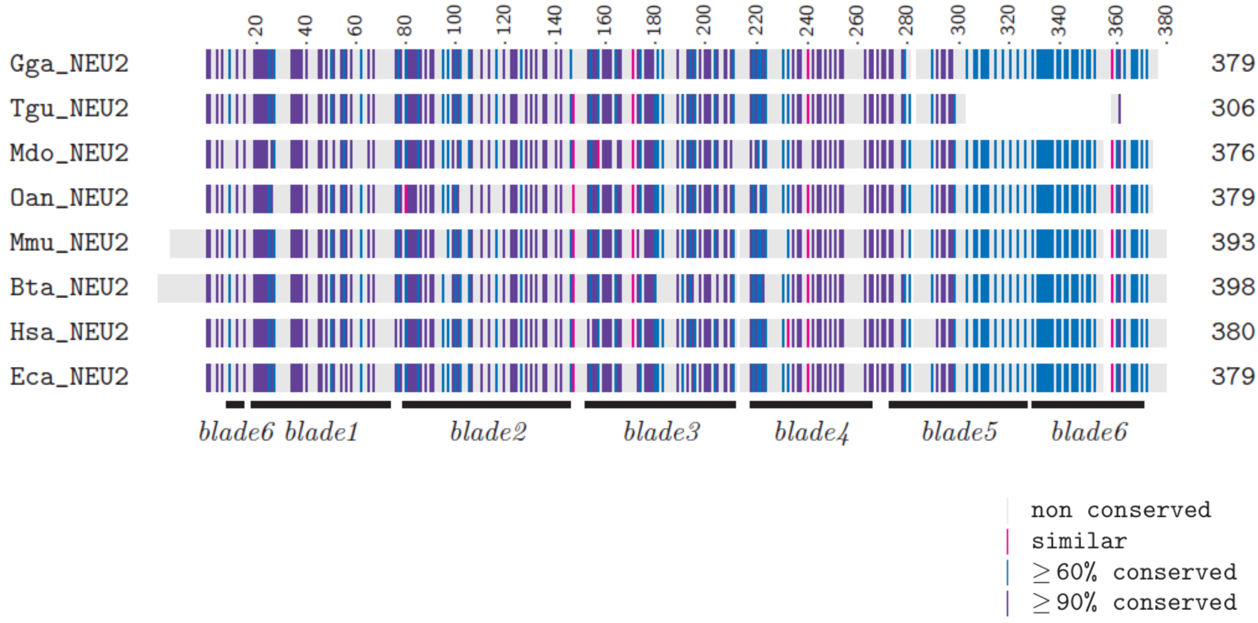

NEU3 Subgroup Alignment. Multiple alignment of NEU3 protein sequences identified. Alignment is elaborated with TexShade and shown in fingerprint style. The regions corresponding to the 6 blades that compose the sialidase β-propeller structure are indicated below the alignment.

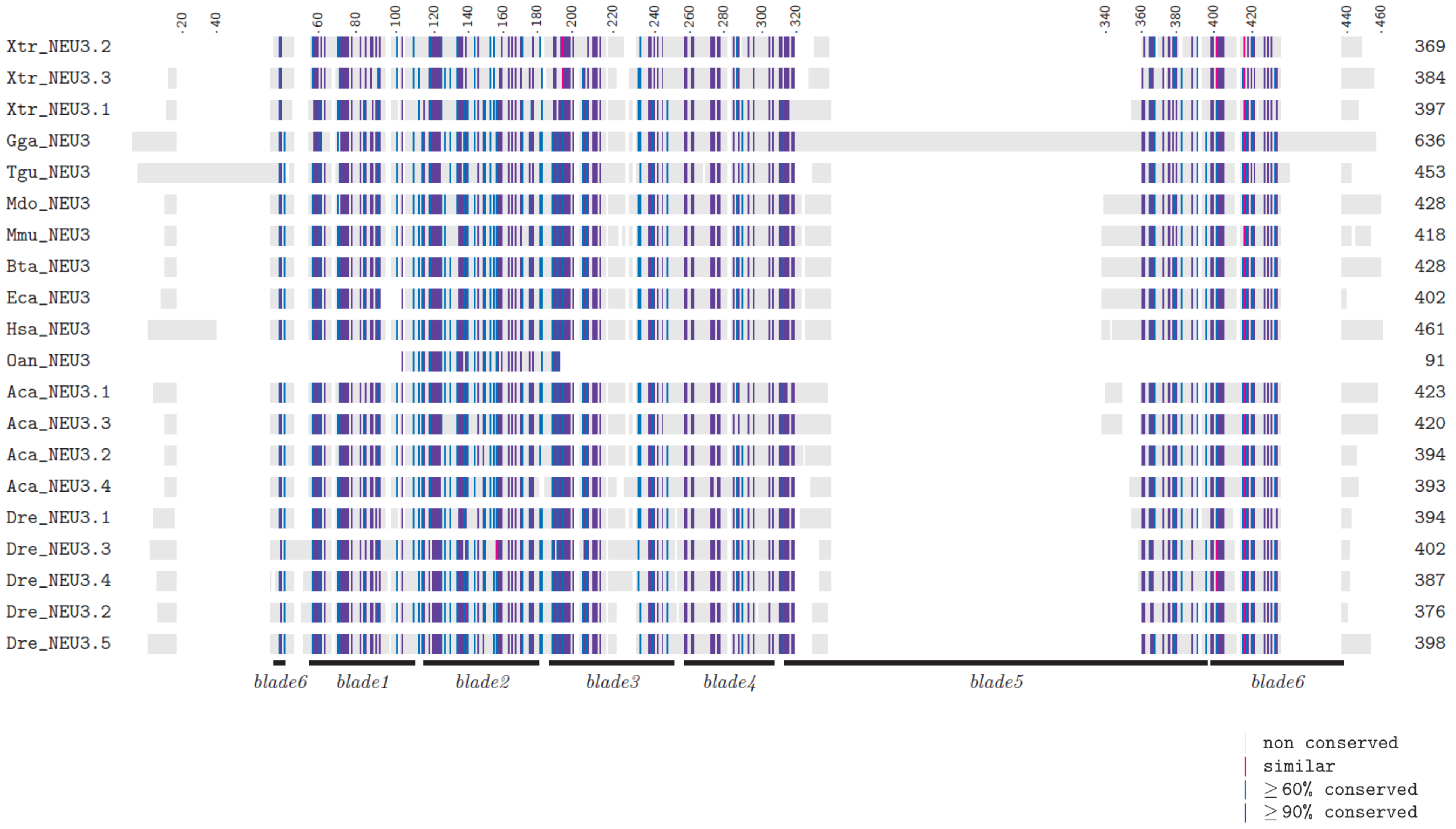

NEU4 Subgroup Alignment. Multiple alignment of NEU4 protein sequences identified. Alignment is elaborated with TexShade and shown in fingerprint style. The regions corresponding to the 6 blades that compose the sialidase β-propeller structure are indicated below the alignment.

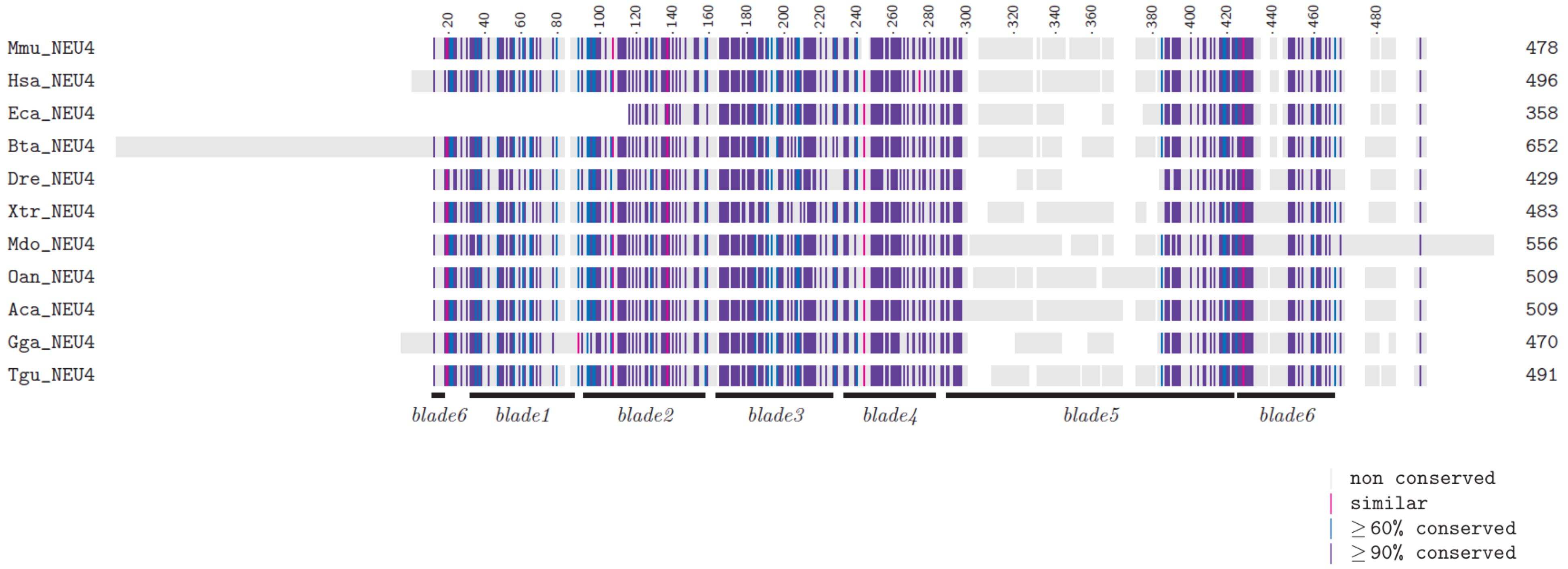

NEU5 Subgroup Alignment. Multiple alignment of NEU3/4 protein sequences identified. Alignment is elaborated with TexShade and shown in fingerprint style. The regions corresponding to the 6 blades that compose the sialidase β-propeller structure are indicated below the alignment.

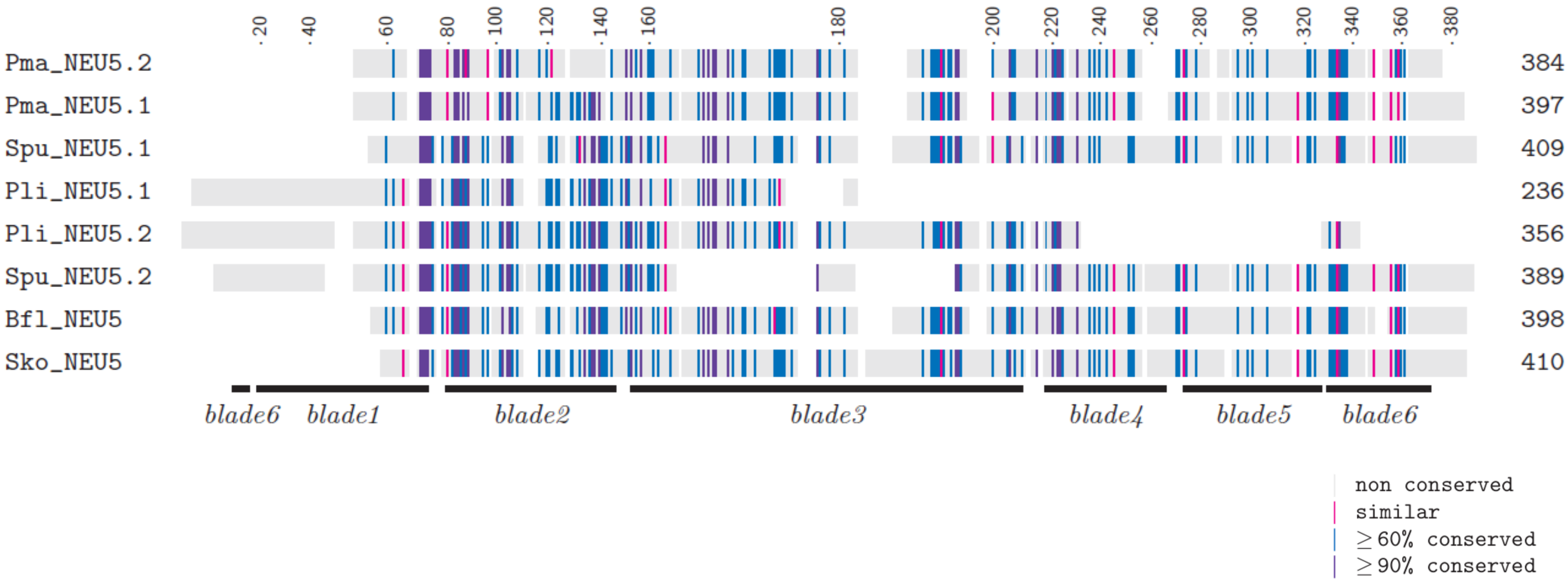

Supplement: Subgroup Alignments S1 — Multiple alignments of NEU1–4 protein sequences subgroups identified. Alignment is elaborated with TexShade and shown in fingerprint style. The regions corresponding to the 6 blades that compose the sialidase β- propeller structure are indicated below the alignment. (PDF) [file pone.0044193.s004.pdf]
