# Supplementary material for: New Insights on the Sialidase Protein Family Revealed by a Phylogenetic Analysis in Metazoa
Source: PLoS One. 2012 Aug 30;7(8):e44193. doi: 10.1371/journal.pone.0044193 (PMC3431349; doi:10.1371/journal.pone.0044193)
Supplement: Table S1 — Information on the 87 sialidase sequences. (PDF) [file pone.0044193.s005.pdf]

Table S1. Information on the 87 sialidase sequences.

Name	Protein data			mRNA data					CDS	Notes
	based on GenBank	full seq GenBank	aa	based on GenBank	full seq GenBank	Genomic location	bp mRNA	bp gDNA	position in mRNA	
Aca_NEU1			405	FG678104, FG678056, FG689267	BK008524*	chr2:194,100,430-194,126,131	1863	25702	76-1293	
Bfl_NEU1.1	XP_002609278		383	FE554285, FE554284, XM_002609232	BK008525*	chrUn:806,076,646-806,080,557	1409	3912	1-1149	No 5' UTR
Bfl_NEU1.2		XP_002609277	391		XM_002609231	chrUn:431,815,200-431,817,995	1176	2796	1-1176	No 3'-5' UTR
Bfl_NEU1.3	XP_002609276		393	BW697076, BW715757, XM_002609230	BK008526*	chrUn:806,084,452-806,088,269	1811	3818	330-1511	
Bfl_NEU1.4	XP_002609274		428	BW774419, BW787406, XM_002609228	BK008527*	chrUn:806,091,547-806,094,731	1413	3185	127-1413	No 3' UTR
Bta_NEU1		NP_001077111	415		NM_001083642	Chr21:27,240,454-27,244,415	1887	3962	105-1342	
Dre_NEU1		NP_001038374	383		NM_001044909	Chr19:27,295,873-27,309,312	1553	13440	69-1220	
Eca_NEU1		XP_001491874	415		XM_001491824	NC_009163:31,592,203-31,595,538	1248	3336	1-1248	
Gga_NEU1			85	BG710400	BK008528*		256			5'-3' translation frame 2
Hsa_NEU1		NP_000425	415		NM_000434	Chr6:31,826,829-31,830,709	2088	3881	157-1404	
Pma_NEU1.1			352	ti1192803107, ti1208147866, ti1289692347, ti1489798852, ti1384919345, ti1289665445	BK008529*	Contig10309:4,780-13,706	1060	8927	1-1060	No 3'-5' UTR<br>No ATG
Pma_NEU1.2			393	DY795820, EB083151, EB083128	BK008530*	Contig5934:12,777-15,380	1661	2604	1-1182	No 5' UTR<br>No ATG
Mdo_NEU1		XP_001376295	467		XM_001376258	Chr2:268,057,970-268,065,547	1404	7578		No 3'-5' UTR
Mmu_NEU1		NP_035023	409		NM_010893	Chr17:35,068,198..35,074,242	3850	6045	155-1384	
Oan_NEU1		XP_001520154	260		XM_001520104	Contig47300:26-4,809	784	4784		Lack 5' portion – short genomic contig
Pli_NEU1.1			251	AM210073, AM559155, AM204373, AM200846, AM532480, AM562700, AM202052	BK008531*		840		58-840	Lack 3' portion

Pli_NEU1.2			270	AM208985, AM224040, AM537343, AM542735, AM217825	BK008532*		887		78-887	Lack 3' portion
Pli_NEU1.3			243	AM573863, AM545967, AM545850	BK008533*		851		68-796	Contains an early stop codon
Pli_NEU1.4			193	AM538096, AM219458	BK008534*		914		335-914	Lack 3' portion
Sko_NEU1.1		XP_002734437	374	XM_002734391, FE526992, FE533726, FF495426, FF605332, FF603927, FF475996, FF515111, FF495425, FF453465, FF603926, FF533725, FF443118, FF605331, FF637280	BK008535*		1231		64-1183	
Sko_NEU1.2		XP_002738729	436	FF434445, FF433268, FF527859	XM_002738683 BK008536*		1324		14-1324	
Sko_NEU1.3		XP_002734439	366	FF627142,FF627141	XM_002734393 BK008537*		1101		1-1101	
Sko_NEU1.4			204	FF430623	BK008538*		613		1-613	
Spu_NEU1.1		XP_790369	380	CX557315, CD304123, CX679384	XM_785276	Scaffold48154:19,883-29,283	1183	9401	41-1183	
Spu_NEU1.2			380	CD305375	BK008539*	Scaffold14529:27,748-32,249	1138	4502	1-1138	EST extended with GenScan
Spu_NEU1.3			380	AAGJ04036036	BK008540*	Scaffold14529:22,606-24,909	1140	2304	1-1140	
Spu_NEU1.4			378	CX677985, CX681386, CX681859, CX686972, DN581554, DN572782, CD290017	BK008541*	Scaffold14529:14,895-17,634	1134	2740	1-1134	Final seq predicted by GenScan
Ssc_NEU1		NP_001095292	416		NM_001101822	Chr7:27,775,646-27,780,585	1932	4940	139-1389	
Olo_NEU1.1			277	AM764102	BK008542*		834		1-834	Protein translated from EST (frame2)
Olo_NEU1.2			284	AM763655, AM762908	BK008543*		854		1-854	Lack 3' 5' ends
Xla_NEU1		NP_001086082	398		NM_001092613		2413		180-1376	
Mbr_NEU1.1		XP_001749157	327		XM_001749105	NW_001865065.1:324,051-325,646	984	1596	1-984	
Mbr_NEU1.2		XP_001749247	445		XM_001749195	NW_001865065.1:408,023-409,897	1338	1875	1-1338	
Mbr_NEU1.3		XP_001743349	357		XM_001743297	NW_001865040.1:882,121-883,543	1074	1423	1-1074	

Mbr_NEU1.4		XP_001748984	439		XM_001748932	NW_001865063.1:554,033-556,541	1320	2509	1-1320	
Mbr_NEU1.5		XP_001749953	394		XM_001749901	NW_001865071.1:394,382-395,722	1185	1341	1-1185	
Kbr_NEU1.1			427	EX979741, EX979740	BK008544*		1450		45-1328	
Kbr_NEU1.2			368	EX870849, EX870848	BK008545*		1150		38-1144	
Svo_NEU1.1			264	GH183667	BK008546*		796		1-796	
Svo_NEU1.2			239	GH181999	BK008547*		719		1-719	Contains a stop codon at position 343
Bta_NEU2		XP_003585408	379		XM_003585360	chr3:113,369,455-113,371,967	1140	2513	1-1140	
Eca_NEU2		XP_001499172	379		XM_001499122	chr6:19,996,515-19,998,937	1140	2423	1-1140	
Gga_NEU2		XP_001231585	379	BM440053	XM_001231584	chr9:2,053,986-2,061,693	1380	7708	65-1204	
Tgu_NEU2			306	ABQF01030247	BK008548*	chr9:7,038,424-7,041,044	921	2621	1-921	
Hsa_NEU2		NP_005374	380		NM_005383	Chr2:233,897,382-233,899,767	1143	2386	1-1143	
Mmu_NEU2		NP_001153635	393		NM_001160163	Chr1:89,470,602-89,494,420	1722	23819	172-1353	
Oan_NEU2		XP_001520932	379		XM_001520882	Contig593:337,062-341,533	1140	4472	1-1140	
Mdo_NEU2		XP_001373563	376		XM_001373526	chr2:520,608,623-520,611,258	1131	2636	1-1131	
Aca_NEU3.1			423	AAWZ02028956	BK008549*	Scaffold_720:421,949-424,253	1276	2305	1-1276	
Aca_NEU3.2			394	AAWZ02028955	BK008550*	Scaffold_720:390,919-401,396	1185	10478	1-1185	
Aca_NEU3.3			420	AAWZ02028954	BK008551*	Scaffold_720:376,486-381,173	1263	4688	1-1263	
Aca_NEU3.4			393	AAWZ02028954	BK008552*	Scaffold_720:349,569-353,958	1182	4390	1-1182	
Bta_NEU3		NP_776547	428		NM_174122	Chr15:54,002,176..54,016,703	3008	14528	259-1545	
Dre_NEU3.1		NP_001073133	394		NM_001079665	Chr21:20,743,670-20,746,642	1599	2973	120-1304	
Dre_NEU3.2		NP_001003644	376		NM_001003644	Chr21:20,748,581-20,751,163	1470	2583	168-1298	
Dre_NEU3.3		NP_001071006	402		NM_001077538	Chr21:20,755,234..20,760,330	2454	5097	141-1349	
Dre_NEU3.4		NP_001103193	387		NM_001109723	Chr21:20,764,182..20,765,510	1164	1329	1-1164	
Dre_NEU3.5		NP_001103203	398		NM_001109733	Chr21:20,769,846..20,771,192	1197	1347	1-1197	
Eca_NEU3		XP_001917436	457		XM_001917401	Chr7:69,058,001-69,065,825	1347	7825	1-1347	
Gga_NEU3		ADJ19117	636		GQ365760	chr1:200,650,939-200,656,547	2421	5609	58-1968	
Hsa_NEU3		NP_006647	461		NM_006656	Chr11:74,699,950..74,718,743	2748	18794	157-1542	
Mdo_NEU3		XP_001366978	428		XM_001366941	Chr4:343,921,177-343,932,992	1287	11816	1-1287	

Mmu_NEU3		NP_057929	418		NM_016720	Chr7:106,959,949-106,976,927	3341	16979	266-1522	
Oan_NEU3		XP_001519053	91		XM_001519003	Contig102757:134-406	274	274	1-274	
Tgu_NEU3	XP_002187487		453	XM_002187451, FE739130, FE735002, DV947363, FE725462, FE729607, DV949468, DV960612, CK306916, FE716748, FE736405, DV961601, CK302608, FE720029, FE731169, FE725024, DV961497, FE718438	BK008553*	chr1:97,816,568-97,819,570	1799	3003	1-1364	No ATG
Xtr_NEU3.1			397	CX815533, EL815973, EL834464, EL835027, EL815393, EL835028, EL815974	BK008554*	scaffold_982:187,281-193,921	1688	6641	80-1273	
Xtr_NEU3.2			369	DN082003	BK008555*	scaffold_982:214,843-217,118	1604	2276	1-1112	Lack 5' (genomic gap) – EST extended with GenScan prediction
Xtr_NEU3.3			384	DN060804, CX936052, CX935200, CX935199, DN064246	BK008556*	scaffold_982:226,393-230,716	1518	4324	22-1176	
Aca_NEU4	XP_003218401		509	XM_003218353	BK008557*	scaffold_66:27,634-35,120	1530	7487	1-1530	
Bta_NEU4		XP_606439	478		XM_606439	Chr3:126,784,306-126,786,649	1437	2344	1-1437	
Dre_NEU4		NP_001018384	429		NM_001020548	Chr15:2,481,270..2,488,632	1438	7363	89-1378	
Eca_NEU4			358	AAWR02034804, AAWR02034805		chr6:27,041,935-27,043,943	1190	2009	1-1190	Lack 5' – portion between V194-P196 located in genomic gap
Gga_NEU4			470	XR_027015, DR425208, BU302275	BK008558*	chr9:5,790,850-5,794,587	1410	3738	1-1410	XR corrected with EST and BLAT
Hsa_NEU4L		NP_542779	496		NM_080741	Chr2:242,752,030..242,758,739	2370	6710	495-1985	
Mdo_NEU4		XP_001366577	556		XM_001366540	Chr7:253,379,303..253,417,504	1671	38202	1-1671	
Mmu_NEU4		NP_776133	478		NM_173772	Chr1:95,917,070..95,924,911	4524	7842	157-1593	
Oan_NEU4		XP_001513020	509		XM_001512970	Chr7:30,754,106..30,761,025	1530	6920	1-1530	
Tgu_NEU4		XP_002191718	491		XM_002191682	Chr9:4,347,568..4,350,041	1756	2474	1-1476	

Xt_NEU4			493	ES684896	BK008559*	scaffold_1219:86,310-90,525	1452	4216	1-1452	
Bfl_NEU5	XP_002586577		398	XM_002586531, FE578609	BK008560*	chrUn:789,396,952-789,404,346	1213	7395	18-1213	
Pli_NEU5.1			236	AM538356, AM527862, AM538734, AM224764, AM514814, AM566984, AM535942, AM536346, AM513124, AM528522, AM546331, AM539940	BK008561*		819		111-819	
Pli_NEU5.2			356	AM551104, AM597930, AM596712, AM593972, AM597695, AM524175, AM558209, AM222256, AM542232, AM208642, AM509895, AM217487, AM222882, AM516936, AM219616, AM597734, AM226312, AM529851	BK008562*		1343		276-1343	Lack 3' portion
Sko_NEU5		XP_002742125	410	FF490281	XM_002742079		1233		1-1233	
Spu_NEU5.1	XP_788431		410	XM_783338, JT117638	BK008563*	Scaffold19943:1,239-8,566	1227	7328	1-1227	
Spu_NEU5.2	XP_001202482		389	DN790884, DN565442, CD291838, AF122522 XM_001202482	BK008564*	Scaffold70614:3,410-28,453 Scaffold5061:3,449-11,994	1783			Split on 2 different contigs, lack the central part at D169
Pma_NEU5.1			397	FD708713, FD713066, FD708714, FD713067, CO544574, EG023197	BK008565*	Contig39489:1-7,000 Contig32577:9,764-10,817	1384		125-1318	
Pma_NEU5.2			384	ti1433634073, ti1165811163, ti1467939775, ti1484873149		Contig42308:1,454-3,607	1179	2154	1-1179	

Reported sequences are based on accession numbers in the “based on Genbank” column which have been assembled or extended with data from other databases (see Materials and Methods). The GenBank accession number corresponding to the entire sequence is reported in “full seq GenBank” column. New sequences submitted with this work are indicated by * in this column. When determined, the position of the CDS within the mRNA sequence is reported in the “position in mRNA” column.
