# Supplementary material for: New Insights on the Sialidase Protein Family Revealed by a Phylogenetic Analysis in Metazoa
Source: PLoS One. 2012 Aug 30;7(8):e44193. doi: 10.1371/journal.pone.0044193 (PMC3431349; doi:10.1371/journal.pone.0044193)
Supplement: Table S2 — Conservation of the predicted phosphorylatable sites in each sialidase subgroups. (PDF) [file pone.0044193.s006.pdf]

Table S2. Conservation of the predicted phosphorylation sites in each sialidase subgroups.

Sites predicted by NetPhos 2.0	Absolut position	NEU1	NEU2	NEU3	NEU4	NEU5	All NEUs	Binomial test	Fisher's Test NEU1	Fisher's Test NEU2	Fisher's Test NEU3	Fisher's Test NEU4	Fisher's Test NEU5
NEU1 S7	128	10,81%	0,00%	0,00%	0,00%	0,00%	4,82%						
NEU1 T8	129	8,11%	0,00%	0,00%	0,00%	0,00%	3,61%						
NEU3 S14	154	10,81%	0,00%	10,53%	0,00%	0,00%	7,23%						
NEU3 S17	157	10,81%	0,00%	10,53%	0,00%	0,00%	7,23%						
NEU3 S18	158	8,11%	0,00%	5,26%	0,00%	0,00%	4,82%						
NEU3 T22	162	10,81%	12,50%	5,26%	0,00%	0,00%	7,23%						
NEU1 S43	164	13,51%	0,00%	5,26%	9,09%	0,00%	8,43%						
NEU3 S29	169	13,51%	12,50%	21,05%	0,00%	0,00%	12,05%						
NEU3 S30	170	21,62%	12,50%	10,53%	0,00%	0,00%	13,25%						
NEU4 S12	174	18,92%	0,00%	15,79%	27,27%	12,50%	16,87%	7.48E-04					
NEU3 T38	178	2,70%	12,50%	15,79%	9,09%	0,00%	7,23%						
NEU4 T18	188	2,70%	12,50%	15,79%	18,18%	0,00%	8,43%						
NEU1 T76; NEU3 T56; NEU4 T33	233	43,24%	0,00%	68,42%	90,91%	75,00%	54,22%	1.39E-45					
NEU4 S38	241	13,51%	0,00%	10,53%	9,09%	0,00%	9,64%						
NEU1 T85	245	59,46%	75,00%	78,95%	36,36%	25,00%	59,04%	1.65E-52					
NEU1 T89	252	51,35%	75,00%	84,21%	45,45%	12,50%	56,63%	5.28E-49					
NEU3 S79	264	0,00%	12,50%	63,16%	0,00%	25,00%	18,07%	8.32E-05			1.20E-03		
NEU2 S43; NEU3 T80; NEU4 S57	265	35,14%	62,50%	89,47%	90,91%	62,50%	60,24%	2.72E-54					
NEU1 S100	266	83,78%	25,00%	10,53%	0,00%	62,50%	48,19%	2.13E-37	1.45E-05				
NEU1 S101	267	43,24%	0,00%	0,00%	0,00%	25,00%	21,69%	7.23E-08					
NEU1 S102	281	45,95%	12,50%	10,53%	0,00%	0,00%	24,10%	4.63E-10					
NEU1 S114	301	94,59%	0,00%	0,00%	0,00%	0,00%	42,17%	9.88E-30	8.81E-18				

NEU4 T72	302	45,95%	50,00%	10,53%	72,73%	0,00%	37,35%	5.58E-24					
NEU2 Y59	303	0,00%	100,00%	5,26%	0,00%	0,00%	10,84%			1.59E-06			
NEU4 S77	312	81,08%	0,00%	57,89%	81,82%	0,00%	60,24%	2.72E-54					
NEU5 T68	327	5,41%	37,50%	36,84%	9,09%	12,50%	16,87%	7.48E-04					
NEU3 T145	364	5,41%	100,00%	100,00%	27,27%	100,00%	48,19%	2.13E-37	4.14E-09		3.58E-04		
NEU3 S152; NEU4 T129	371	10,81%	87,50%	47,37%	90,91%	37,50%	39,76%	8.20E-27	8.55E-03				
NEU1 S174	384	97,30%	100,00%	100,00%	100,00%	100,00%	98,80%	2.85E-127					
NEU1 S180; NEU3 S170; NEU4 S147	390	97,30%	100,00%	94,74%	90,91%	100,00%	96,39%	5.38E-121					
NEU1 T183; NEU2 S138; NEU4 S150	393	18,92%	25,00%	5,26%	36,36%	0,00%	16,87%	7.48E-04					
NEU1 S188; NEU3 T178; NEU5 T136	398	75,68%	100,00%	100,00%	100,00%	87,50%	87,95%	2.94E-102					
NEU2 S173	449	2,70%	87,50%	0,00%	0,00%	0,00%	9,64%			1.06E-04			
NEU4 T190	456	18,92%	0,00%	31,58%	81,82%	25,00%	28,92%	8.89E-15					
NEU5 S177	462	5,41%	12,50%	0,00%	9,09%	12,50%	6,02%						
NEU4 S206	492	18,92%	0,00%	26,32%	90,91%	62,50%	32,53%	1.40E-18					
NEU3 S233; NEU4 S209	495	5,41%	0,00%	57,89%	81,82%	62,50%	32,53%	1.40E-18	9.17E-03				
NEU5 Y196	499	21,62%	0,00%	100,00%	90,91%	62,50%	50,60%	1.31E-40			1.22E-03		
NEU1 S239	506	83,78%	100,00%	73,68%	63,64%	75,00%	79,52%	5.09E-86					
NEU1 S244	512	18,92%	12,50%	5,26%	0,00%	0,00%	10,84%						
NEU1 Y251	519	43,24%	0,00%	0,00%	0,00%	0,00%	19,28%	8.56E-06	1.41E-03				
NEU5 S219	533	5,41%	62,50%	100,00%	100,00%	37,50%	48,19%	2.13E-37	4.14E-09		3.58E-04		
NEU1 Y268	539	27,03%	0,00%	0,00%	0,00%	12,50%	13,25%						
NEU3 T265	542	0,00%	0,00%	42,11%	27,27%	0,00%	13,25%						
NEU2 S238; NEU4 S255	556	10,81%	100,00%	78,95%	100,00%	37,50%	49,40%	5.40E-39	1.45E-06				
NEU5 S252	567	10,81%	0,00%	0,00%	0,00%	25,00%	7,23%						
NEU1 S295; NEU2 S248; NEU3 S289; NEU4 S265; NEU5 S254	569	100,00%	100,00%	78,95%	90,91%	100,00%	93,42%	2.00E-97					

NEU1 T301; NEU4 S271	575	87,50%	0,00%	10,53%	36,36%	100,00%	52,63%	2.13E-37					
NEU1 T308	582	50,00%	0,00%	10,53%	36,36%	100,00%	36,84%	6.76E-20					
NEU4 S280; NEU5 S269	585	15,63%	12,50%	5,26%	27,27%	16,67%	14,47%						
NEU2 S281	620	0,00%	25,00%	5,26%	0,00%	0,00%	3,95%						
NEU2 S284	624	0,00%	25,00%	5,26%	54,55%	0,00%	11,84%						
NEU4 S307	643	0,00%	0,00%	0,00%	18,18%	0,00%	2,63%						
NEU4 S309	645	0,00%	0,00%	5,26%	36,36%	0,00%	6,58%						
NEU4 S314	650	0,00%	0,00%	0,00%	18,18%	0,00%	2,63%						
NEU2 S288	662	0,00%	25,00%	10,53%	0,00%	0,00%	5,26%						
NEU3 S332	666	0,00%	0,00%	10,53%	0,00%	0,00%	2,63%						
NEU3 S333	667	0,00%	0,00%	5,26%	0,00%	0,00%	1,32%						
NEU3 S343	677	0,00%	0,00%	15,79%	18,18%	0,00%	6,58%						
NEU3 S346	681	0,00%	0,00%	31,58%	9,09%	0,00%	9,21%						
NEU3 S347	682	0,00%	0,00%	26,32%	9,09%	0,00%	7,89%						
NEU3 T356	734	0,00%	0,00%	36,84%	9,09%	33,33%	13,16%						
NEU4 S386	736	0,00%	0,00%	31,58%	54,55%	0,00%	15,79%						
NEU5 S308	814	0,00%	0,00%	0,00%	0,00%	16,67%	1,32%						
NEU3 T368	826	6,25%	87,50%	94,74%	63,64%	33,33%	47,37%	3.19E-31	9.10E-07		1.16E-03		
NEU3 S369	827	40,63%	0,00%	31,58%	54,55%	16,67%	34,21%	2.74E-17					
NEU2 S301	830	28,13%	50,00%	10,53%	27,27%	0,00%	23,68%	7.23E-08					
NEU1 T345	837	81,25%	0,00%	0,00%	0,00%	50,00%	38,16%	3.10E-21	3.27E-06				
NEU4 S409	841	0,00%	0,00%	0,00%	100,00%	0,00%	14,47%					4.32E-10	
NEU3 T383; NEU4 S411	843	9,38%	12,50%	63,16%	45,45%	16,67%	28,95%	2.29E-12					
NEU1 S349	844	84,38%	0,00%	0,00%	0,00%	66,67%	40,79%	5.58E-24	4.59E-06				
NEU4 S417	849	46,88%	12,50%	26,32%	100,00%	0,00%	42,11%	2.19E-25					
NEU1 S355	850	84,38%	0,00%	0,00%	0,00%	66,67%	40,79%	5.58E-24	4.59E-06				
NEU4 S429	863	68,75%	0,00%	68,42%	100,00%	83,33%	67,11%	4.26E-56					
NEU1 Y370; NEU4 Y431;	865	84,38%	87,50%	100,00%	100,00%	100,00%	92,11%	4.52E-95					

NEU5 Y353													
NEU1 S379	874	25,00%	0,00%	10,53%	9,09%	50,00%	18,42%	7.48E-04					
NEU2 S347	886	6,25%	87,50%	0,00%	9,09%	16,67%	14,47%			4.20E-03			
NEU2 Y354; NEU4 Y451	893	71,88%	50,00%	5,26%	72,73%	66,67%	52,63%	2.13E-37					
NEU4 T457	902	21,88%	0,00%	47,37%	81,82%	16,67%	34,21%	2.74E-17					
NEU4 S458	903	53,13%	12,50%	5,26%	45,45%	33,33%	34,21%	2.74E-17					
NEU2 Y359; NEU4 Y459	904	43,75%	87,50%	26,32%	100,00%	66,67%	53,95%	5.40E-39					
NEU1 S410; NEU3 T436; NEU5 S388	913	40,63%	87,50%	63,16%	100,00%	83,33%	63,16%	9.58E-51					
NEU4 S479	1010	0,00%	0,00%	0,00%	36,36%	0,00%	5,26%						
NEU3 S450	1018	0,00%	0,00%	15,79%	0,00%	0,00%	3,95%						

For every site predicted by NetPhos2.0 the table reports its position in the global multiple alignments comprising all the 83 sialidase sequences (see Multiple FASTA alignment S1 in Supporting Materials) and the percentage of conservation for the phosphorylation site in the five sialidase subgroups. The percentage of conservation in the entire dataset (83 sequences) is also reported in the “All NEUs” column. Significance of conservation of each site in the global alignments of all sialidase sequences has been tested with a Binomial test, significance of specific conservation in the single sialidase subgroups has been assessed with the Fisher's exact test (see Materials and Methods). Significant corrected p-values (< 0.01) are reported in the table.
