# Supplementary material for: New Insights on the Sialidase Protein Family Revealed by a Phylogenetic Analysis in Metazoa
Source: PLoS One. 2012 Aug 30;7(8):e44193. doi: 10.1371/journal.pone.0044193 (PMC3431349; doi:10.1371/journal.pone.0044193)
Supplement: Table S3 — Conservation of the predicted phosphorylatable sites in mammals, vertebrates and deuterostomes. (PDF) [file pone.0044193.s007.pdf]

Table S3. Conservation of the predicted phosphorylation sites in mammals, vertebrates and deuterostomes.

Sites predicted by NetPhos 2.0	Absolute position	mammals	vertebrates	deuterostomes	early Metazoa	Fisher's mammals	Fisher's vertebrates	Fisher's deuterostomes
NEU1 S7	128	14.29%	5.77%	4.05%	10.00%			
NEU1 T8	129	9.52%	3.85%	2.70%	10.00%			
NEU3 S14	154	14.29%	7.69%	8.11%	0.00%			
NEU3 S17	157	9.52%	7.69%	9.46%	0.00%			
NEU3 S18	158	4.76%	3.85%	5.41%	10.00%			
NEU3 T22	162	9.52%	3.85%	5.41%	20.00%			
NEU1 S43	164	14.29%	5.77%	6.76%	20.00%			
NEU3 S29	169	9.52%	9.62%	9.46%	30.00%			
NEU3 S30	170	9.52%	9.62%	9.46%	40.00%			
NEU4 S12	174	23.81%	21.15%	18.92%	0.00%			
NEU3 T38	178	19.05%	7.69%	5.41%	10.00%			
NEU4 T18	188	9.52%	11.54%	8.70%	12.50%			
NEU1 T76; NEU3 T56; NEU4 T33	233	71.43%	71.15%	64.86%	0.00%			
NEU4 S38	241	4.76%	5.77%	6.76%	40.00%			
NEU1 T85	245	28.57%	21.15%	27.03%	20.00%			
NEU1 T89	252	90.48%	67.31%	55.41%	60.00%			
NEU3 S79	264	23.81%	28.85%	20.27%	0.00%			
NEU2 S43; NEU3 T80; NEU4 S57	265	61.90%	67.31%	66.22%	10.00%			
NEU1 S100	266	38.10%	26.92%	43.24%	70.00%			
NEU1 S101	267	19.05%	15.38%	21.62%	20.00%			
NEU1 S102	281	33.33%	25.00%	24.32%	20.00%			
NEU1 S114	301	28.57%	21.15%	36.49%	90.00%			
NEU4 T72	302	38.10%	36.54%	36.49%	40.00%			
NEU2 Y59	303	23.81%	17.31%	12.16%	10.00%			

NEU4 S77	312	61.90%	61.54%	59.46%	70.00%			
NEU5 T68	327	14.29%	21.15%	16.22%	30.00%			
NEU3 T145	364	52.38%	61.54%	54.05%	0.00%			
NEU3 S152; NEU4 T129	371	57.14%	53.85%	44.59%	0.00%			
NEU1 S174	384	100.00%	100.00%	100.00%	90.00%			
NEU1 S180; NEU3 S170; NEU4 S147	390	100.00%	96.15%	97.30%	90.00%			
NEU1 T183; NEU2 S138; NEU4 S150	393	33.33%	23.08%	16.22%	20.00%			
NEU1 S188; NEU3 T178; NEU5 T136	398	100.00%	100.00%	89.19%	70.00%			
NEU2 S173	449	19.05%	13.46%	9.46%	10.00%			
NEU4 T190	456	28.57%	28.85%	31.08%	20.00%			
NEU5 S177	462	4.76%	1.92%	2.70%	20.00%			
NEU4 S206	492	33.33%	36.54%	29.17%	60.00%			
NEU3 S233; NEU4 S209	495	47.62%	40.38%	32.43%	20.00%			
NEU5 Y196	499	47.62%	61.54%	52.70%	50.00%			
NEU1 S239	506	100.00%	76.92%	78.38%	80.00%			
NEU1 S244	512	19.05%	9.62%	10.81%	10.00%			
NEU1 Y251	519	19.05%	15.38%	18.92%	20.00%			
NEU5 S219	533	71.43%	71.15%	51.35%	20.00%		1.01E-04	
NEU1 Y268	539	28.57%	19.23%	14.86%	0.00%			
NEU3 T265	542	14.29%	21.15%	14.86%	0.00%			
NEU2 S238; NEU4 S255	556	71.43%	71.15%	54.05%	10.00%		6.85E-04	
NEU5 S252	567	0.00%	0.00%	7.25%	10.00%			
NEU1 S295; NEU2 S248; NEU3 S289; NEU4 S265; NEU5 S254	569	100.00%	88.46%	82.43%	100.00%			
NEU1 T301; NEU4 S271	575	62.50%	36.54%	49.28%	70.00%			
NEU1 T308	582	37.50%	17.31%	18.84%	30.00%			

NEU4 S280; NEU5 S269	585	23.81%	11.54%	11.11%	30.00%				
NEU2 S281	620	9.52%	5.77%	4.05%	0.00%				
NEU2 S284	624	19.05%	19.23%	13.51%	0.00%				
NEU4 S307	643	4.76%	3.85%	2.70%	0.00%				
NEU4 S309	645	19.05%	9.62%	6.76%	0.00%				
NEU4 S314	650	4.76%	3.85%	2.70%	0.00%				
NEU2 S288	662	19.05%	7.69%	5.41%	0.00%				
NEU3 S332	666	9.52%	3.85%	2.70%	0.00%				
NEU3 S333	667	4.76%	3.85%	2.70%	0.00%				
NEU3 S343	677	9.52%	9.62%	6.76%	0.00%				
NEU3 S346	681	23.81%	13.46%	9.46%	0.00%				
NEU3 S347	682	19.05%	11.54%	8.70%	0.00%				
NEU3 T356	734	23.81%	21.15%	14.86%	0.00%				
NEU4 S386	736	38.10%	21.15%	14.86%	0.00%				
NEU5 S308	814	0.00%	0.00%	1.45%	0.00%				
NEU3 T368	826	52.38%	67.31%	50.72%	0.00%			2.90E-07	
NEU3 S369	827	19.05%	28.85%	33.33%	37.50%				
NEU2 S301	830	19.05%	19.23%	21.74%	37.50%				
NEU1 T345	837	37.50%	19.23%	33.33%	60.00%				
NEU4 S409	841	23.81%	21.15%	14.86%	0.00%				
NEU3 T383; NEU4 S411	843	43.75%	36.54%	27.54%	37.50%				
NEU1 S349	844	28.57%	19.23%	36.23%	87.50%				
NEU4 S417	849	57.14%	50.00%	44.93%	25.00%				
NEU1 S355	850	28.57%	21.15%	37.68%	62.50%				
NEU4 S429	863	61.90%	69.23%	66.67%	50.00%				
NEU1 Y370; NEU4 Y431; NEU5 Y353	865	100.00%	94.23%	94.20%	87.50%				
NEU1 S379	874	23.81%	21.15%	18.84%	25.00%				

NEU2 S347	886	33.33%	19.23%	16.22%	0.00%			
NEU2 Y354; NEU4 Y451	893	61.90%	48.08%	53.62%	37.50%			
NEU4 T457	902	14.29%	38.46%	34.78%	25.00%			
NEU4 S458	903	47.62%	34.62%	34.78%	25.00%			
NEU2 Y359; NEU4 Y459	904	76.19%	61.54%	57.97%	37.50%			
NEU1 S410; NEU3 T436; NEU5 S388	913	100.00%	75.00%	65.22%	25.00%	2.67E-03		
NEU4 S479	1010	4.76%	7.69%	5.80%	0.00%			
NEU3 S450	1018	14.29%	5.77%	4.35%	0.00%			

For every site predicted by NetPhos2.0 the table reports its position in the global multiple alignments comprising all the 83 sialidase sequences (see Multiple FASTA alignment S1 in Supporting Materials) and the percentage of conservation for the phosphorylation site in the four taxonomic groups as defined in Materials and Methods. Significance of specific conservation in the single taxonomic groups has been assessed with the Fisher's exact test (see Materials and Methods). Significant corrected p-values (< 0.01) are reported in the table.
